# Engineering cofactor metabolism for improved protein and glucoamylase production in *Aspergillus niger*

**DOI:** 10.1186/s12934-020-01450-w

**Published:** 2020-10-23

**Authors:** Yu-fei Sui, Tabea Schütze, Li-ming Ouyang, Hongzhong Lu, Peng Liu, Xianzun Xiao, Jie Qi, Ying-Ping Zhuang, Vera Meyer

**Affiliations:** 1 State Key Laboratory of Bioreactor Engineering, East China University of Science and Technology, Shanghai, 200237 People’s Republic of China; 2 Chair of Applied and Molecular Microbiology, Institute of Biotechnology, Technische Universität Berlin, Straße des 17. Juni 135, 10623 Berlin, Germany; 3 Department of Biology and Biological Engineering, Chalmers University of Technology, Kemivägen 10, 412 96 Gothenburg, Sweden

**Keywords:** *Aspergillus niger*, NADPH, Genetic engineering, CRISPR/Cas9, Tet-on, Metabolic engineering, Chemostat, Glucoamylase

## Abstract

**Background:**

Nicotinamide adenine dinucleotide phosphate (NADPH) is an important cofactor ensuring intracellular redox balance, anabolism and cell growth in all living systems. Our recent multi-omics analyses of glucoamylase (GlaA) biosynthesis in the filamentous fungal cell factory *Aspergillus niger* indicated that low availability of NADPH might be a limiting factor for GlaA overproduction.

**Results:**

We thus employed the Design-Build-Test-Learn cycle for metabolic engineering to identify and prioritize effective cofactor engineering strategies for GlaA overproduction. Based on available metabolomics and ^13^C metabolic flux analysis data, we individually overexpressed seven predicted genes encoding NADPH generation enzymes under the control of the Tet-on gene switch in two *A. niger* recipient strains, one carrying a single and one carrying seven *glaA* gene copies, respectively, to test their individual effects on GlaA and total protein overproduction. Both strains were selected to understand if a strong pull towards *glaA* biosynthesis (seven gene copies) mandates a higher NADPH supply compared to the native condition (one gene copy). Detailed analysis of all 14 strains cultivated in shake flask cultures uncovered that overexpression of the *gsdA* gene (glucose 6-phosphate dehydrogenase), *gndA* gene (6-phosphogluconate dehydrogenase) and *maeA* gene (NADP-dependent malic enzyme) supported GlaA production on a subtle (10%) but significant level in the background strain carrying seven *glaA* gene copies. We thus performed maltose-limited chemostat cultures combining metabolome analysis for these three isolates to characterize metabolic-level fluctuations caused by cofactor engineering. In these cultures, overexpression of either the *gndA* or *maeA* gene increased the intracellular NADPH pool by 45% and 66%, and the yield of GlaA by 65% and 30%, respectively. In contrast, overexpression of the *gsdA* gene had a negative effect on both total protein and glucoamylase production.

**Conclusions:**

This data suggests for the first time that increased NADPH availability can indeed underpin protein and especially GlaA production in strains where a strong pull towards GlaA biosynthesis exists. This data also indicates that the highest impact on GlaA production can be engineered on a genetic level by increasing the flux through the pentose phosphate pathway (*gndA* gene) followed by engineering the flux through the reverse TCA cycle (*maeA* gene). We thus propose that NADPH cofactor engineering is indeed a valid strategy for metabolic engineering of *A. niger* to improve GlaA production, a strategy which is certainly also applicable to the rational design of other microbial cell factories.
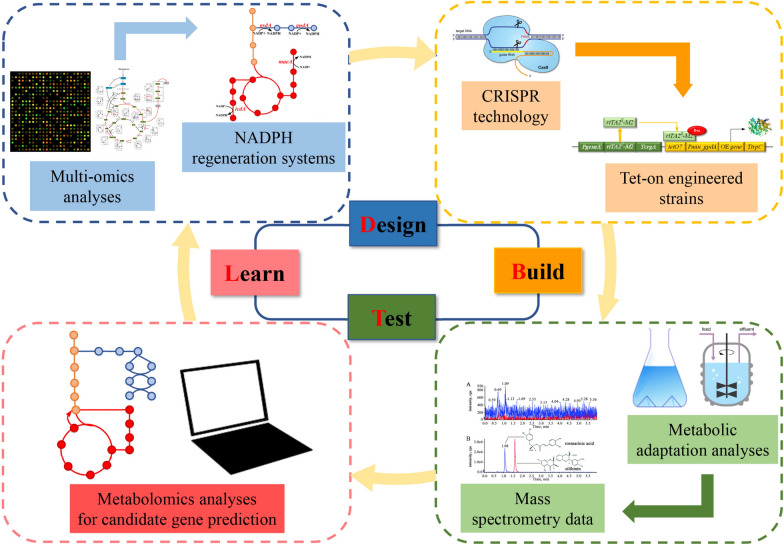

## Background

The filamentous fungus *Aspergillus niger* is one of the main cell factories used nowadays in the industry for homologous or heterologous protein production due to its extraordinary ability for protein expression and secretion [1–3]. The Design-Build-Test-Learn (DBTL) cycle is an increasingly adopted systematic metabolic engineering strategy to achieve the desired outcome through reconstructing heterologous metabolic pathways or rewiring native metabolic activities [4, 5]. Rational strain development of cell factories can be improved by the iterative application of the DBTL cycles, which not only contributes to the optimization of biomanufacturing processes, it is also advantageous to build a complete metabolic model of engineered cells to deepen our understanding of cellular metabolism. Noteworthy, the advance of genetic engineering has speeded up the DBTL cycle of metabolic engineering [6]. For the cell factory *A. niger*, several genetic approaches have proven their potency to improve its enzyme producing capability, including protein carrier approaches, tunable Tet-on driven gene expression, and morphology engineering, to name but a few [1, 7–9]. However, the impact of cofactor engineering, i.e., the rebalance of the intracellular redox status, on protein production has not been systematically studied in *A. niger*.

NADPH is a limiting factor for the biosynthesis of amino acids that are the building blocks of proteins. For instance, 3 mol and 4 mol of NADPH is required for producing 1 mol of arginine and lysine, respectively [10]. Thus, adequate cytosolic NADPH supply is indispensable to maintain the intracellular redox balance and serves as a driving force for efficient amino acid biosynthesis [11]. NADPH also provides the main anabolic reducing power for biomass growth, lipid formation, and also for natural product biosynthesis [12]. Indeed, cofactor engineering has been reported to improve productivities in the bacterial cell factories *Escherichia coli* [13, 14], and *Corynebacterium glutamicum*, as well as in the yeast cell factory *Yarrowia lipolytica* [15]. Two common strategies have mainly been employed to optimize the availability of NADPH. One is to activate the enzyme activities of NAD(H) kinases (EC 2.7.1.86, EC 2.7.1.23) which are used to obtain NADPH or NADP + through phosphorylation of NADH and NAD + , respectively. The other is to modulate the expression strength of typical NADPH generating enzymes of the glycolytic pathway, the pentose phosphate pathway or the citric acid cycle. These include glucose-6-phosphate dehydrogenase (G6PDH), 6-phospho-gluconate dehydrogenase (6PGDH), NADP-dependent isocitrate dehydrogenase (NADP-ICDH), and NADP-dependent malic enzyme (NADP-ME) [16, 17]. Notably, heterologous protein expression in *Pichia pastoris* and *A. niger* can be triggered through boosted carbon flux to the pentose phosphate pathway (PPP), a catabolic pathway also known to produce NADPH [18, 19]. This suggests that central carbon metabolism may have evolved to ensure the production of cellular components under the balance of energy production and consumption [4]. In agreement, the metabolic flux through the PPP increased by 15–26% compared to the parental strains when GlaA was overproduced in *A. niger* [20] or the enzyme amylase overproduced in *A. oryzae* [21].

In the past two decades, extensive studies have focused on engineering a high flux through the PPP in *E. coli*, *C. glutamicum*, *A. nidulans,* and *A. niger* [11, 22–25]. A block of the glycolytic pathway by down-regulating the *pgi* gene encoding a phosphoglucose isomerase was one successful strategy in *C. glutamicum* [11]. In order to elevate the NADPH pool originating from the PPP in *A. niger*, the *gsdA* gene (glucose 6-phosphate dehydrogenase), the *gndA* gene (6-phosphogluconate dehydrogenase) and the *tktA* gene (transketolase) were individually overexpressed in *A. niger*. Strong overexpression of *gndA* led to a nine-fold increase in intracellular NADPH concentration, while *gsdA* and *tktA* affected the NADPH level only weakly [25]. However, any correlation between the NADPH supply and enzyme overproduction remained unclear.

Irrespective of the importance of the PPP for NADPH regeneration, an efficient carbon economy is only guaranteed when the carbon flux enters the glycolytic pathway (Embden-Meyerhoff-Parnass pathway, EMP) instead of the PPP because the PPP releases one carbon as CO_2_ when oxidizing 1 mol of hexose. Takeno et al. [26] thus substituted the endogenous NAD-dependent glyceraldehyde-3-phosphate dehydrogenase (GAPDH) in the EMP in *C. glutamicum* with a heterologous NADP-dependent GAPDH, leading to 2 mol of NADPH generation instead of 2 mol NADH from 1 mol of hexose. This genetic modification provoked a substantial improvement in the yield of L-lysine production by 70–120%. Similar strategies also have been followed to overproduce ethanol in the yeast *Saccharomyces cerevisiae* [27] or lycopene and ε-caprolactone in the bacterium *Clostridium acetobutylicum* [28]. Likewise, cytosolic NADP-ME has been shown to positively affect lipid accumulation in oleaginous fungi [29, 30].

**Fig. 1:**
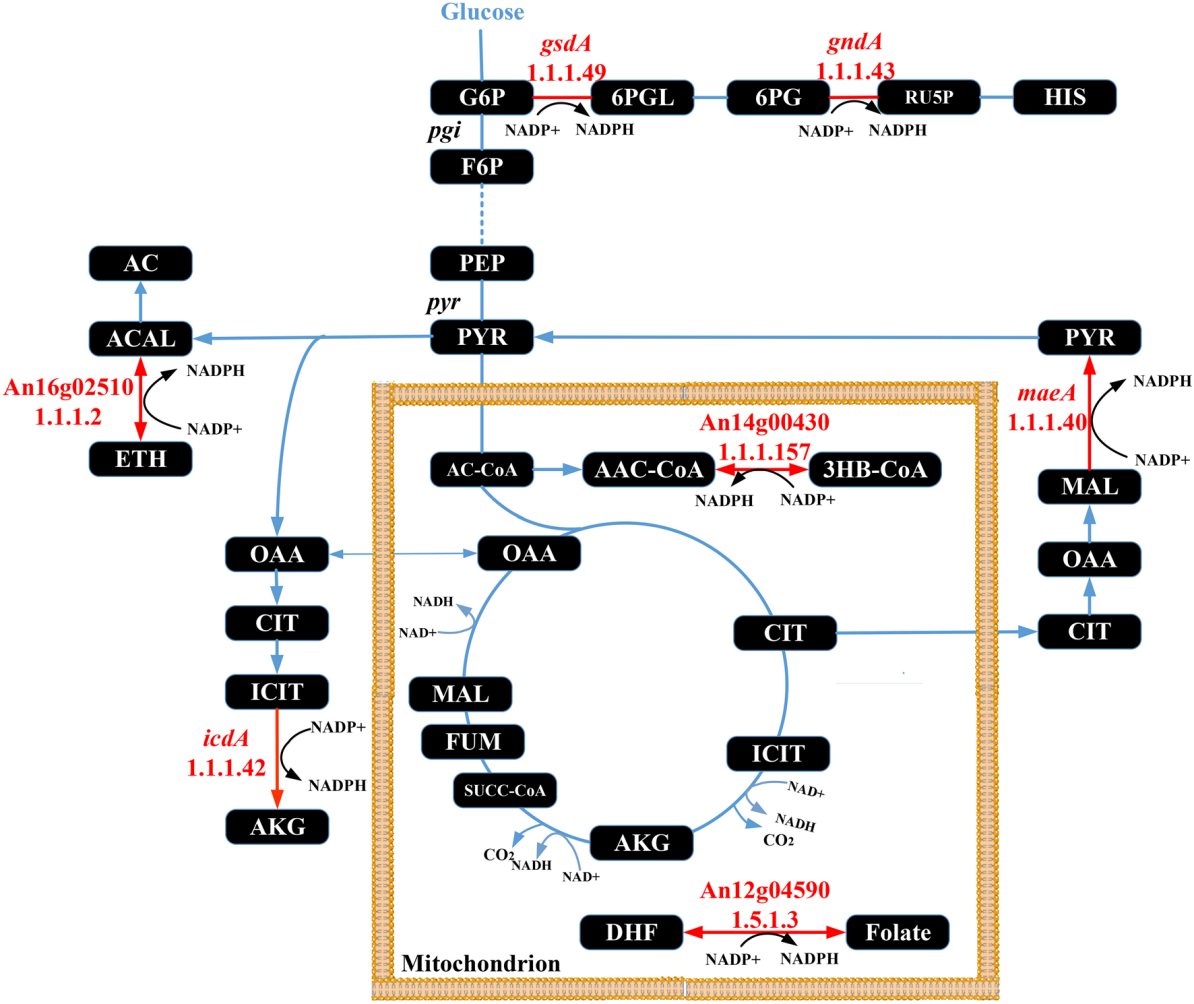
Pathway map highlighting all seven genes modified during this study in red. The cytosolic glycolytic pathway, the pentose phosphate pathway and the mitochondrially located citric acid cycle are shown

As summarized above, a wealth of metabolomic and fluxomic data in *A. niger* demonstrated that strains adapted to protein overproducing conditions channel a higher carbon flux through the PPP. However, cofactor engineering has not been considered yet or performed in *A. niger* to guide enzyme overproduction. We thus mined our recently published genome-scale metabolic network model (GSMM) developed for the *A. niger* protein producing reference strain CBS 513.88 [31]. This iHL1210 model identified the involvement of NADPH in 173 intracellular redox reactions in *A. niger*, including 49 NADPH generating reactions [31]. Notably, the GSMM did not predict any NADPH/NADP + shuttle in the mitochondrial membrane, and we thus concluded that any mitochondrial NADPH is unlikely to become directly consumed by cytosolic amino acid biosynthesis. Overall, the GSMM predicted that seven potential NADPH generating enzymes are of importance for GlaA production in *A. niger* (Table 1, Fig. 1): two enzymes of the cytosolic PPP (glucose-6-phosphate dehydrogenase, G6PDH; 6-phosphogluconate dehydrogenase 6PGDH), two cytosolic NADP-dependent enzymes (NADP-ICDH and NADP-ME) and three uncharacterized open reading frames (An12g04590, An14g00430, An16g02510). An12g04590, An14g00430 show high homology to NADP + oxidoreductases, and An16g02510 displays homology to alcohol dehydrogenases. In order to evaluate whether the model prediction is strain-dependent, we individually overexpressed all seven candidate genes in two *A. niger* host strains. Strain AB4.1 produces native levels of GlaA as it carries one *glaA* gene copy, and strain B36 is a derivative thereof, carrying seven *glaA* gene copies and is thus a high-yield GlaA producing strain [32]. All 14 strains were first investigated in shake flask-level cultivations. Based on the data gained, three engineered strains were selected for chemostat cultivations to decipher the association among genetic perturbation, NADPH availability, and GlaA production in *A. niger*.

**Table 1:** GSMM-predicted NADPH producing reactions in *A. niger*

Rn name	Rn description	Formula	Gene
R25	Glucose 6-phosphate-dehydrogenase (*gsdA*)	G6P + NADP = > D6PGL + NADPH + H	An02g12140
R27	Phosphogluconate dehydrogenase (*gndA*)	6PGC + NADP = > Ru5P + CO_2_ + NADP	An11g02040
R36	Isocitrate dehydrogenase (*icdA*) (NADP +)	ICIT[m] + NADP[m] = > AKG[m] + CO_2_[m] + NADPH[m]	An02g12430
R38	Isocitrate dehydrogenase (NADP +)	ICIT + NADP = > AKG + CO_2_ + NADPH	An02g12430
R55	Malic enzyme (NADP-specific) (*maeA*)	MAL + NADP = > PYR + CO_2_ + NADPH	An05g00930
R57	Malic enzyme (NADP-specific)	MAL[m] + NADP [m] = > PYR [m] + CO_2_[m] + NADPH [m]	An05g00930
R110	(S)-3-Hydroxybutanoyl-CoA: NADP + oxidoreductase	3HBCoA[m] + NADP [m] < = > AACCoA[m] + NADPH [m] +H[m]	An14g00430
R125	dihydrofolate:NADP + oxidoreductase	NADP [m] + DHF[m] < = > NADPH [m] + FOLATE[m]	An12g04590
R190	Alcohol dehydrogenase	ETH + NADP < = > ACAL + NADPH + H	An16g02510

[m] reactions in the mitochondrion; Rn, reaction. All reactions listed here were predicted in Lu et al. [31]

## Results

### Strain generation using CRISPR/Cas9 technology and the synthetic Tet-on gene switch

In order to compare the effect of the seven selected genes on GlaA production in an *A. niger* strain carrying one *glaA* (AB4.1) or seven *glaA* (B36) gene copies, we first had to ensure that the introduced genetic modifications would allow us to directly compare the observed phenotypes. This required that the introduced genes would be under the same genetic control and furthermore introduced at the same genomic locus in both recipient strains. We thus decided to integrate an additional copy of all candidate genes under the control of the strong and tunable Tet-on gene switch into the *pyrG* locus of *A. niger*. This gene switch is inducible by the addition of doxycycline (DOX) to the culture medium, is tight in the absence of DOX and metabolism-independent in *A. niger* [33]. It has furthermore been shown to strongly induce gene expression up to levels above the glucoamylase gene, which is one of the highest expressed genes in *A. niger* [33–35].

Strain AB4.1 is a uridine-auxotroph due to a defective *pyrG* gene, a locus that is perfectly suited for gene targeting and screening purposes. The introduction of an intact *pyrG* copy at this locus occurs efficiently and confers uridine prototrophy in *A. niger* [36]. However, strain B36 does carry an intact *pyrG* gene [37]. We thus first mutated the *pyrG* locus in this strain in order to apply the same gene targeting strategy for all seven genes in both recipient strains. We edited the *pyrG* gene in B36 by following a CRISPR/Cas9 strategy that employed ribonucleoprotein particles. This approach was first published for the penicillin producer *Penicillium chrysogenum* [38] and has later been successfully established in other fungal cell factories [39]. As explained in detail in Additional file 1: Fig. S1, this approach enabled us to obtain a derivative of B36, strain YS20.2, which carries a 195 bp deletion within the *pyrG* ORF and is therefore unable to grow on medium lacking uridine or uracil. Both recipient strains, AB4.1 and YS20.2, were eventually used to integrate Tet-on driven candidate genes at the *pyrG* locus (for details see Materials and Methods and Additional file 1: Fig. S2). Respective genetic modifications were proven by PCR and Southern blot analyses (Additional file 1: Figs. S3, S5, S6, S7). All 14 strains obtained are summarized in Table 3. We finally also decided to delete the native ORFs of An14g00430 and An16g02510 in their respective Tet-on driven overexpression strains in order to analyze their deletion phenotypes.

### The impact of NADPH engineering on GlaA production is strain-dependent

**Fig. 2:**
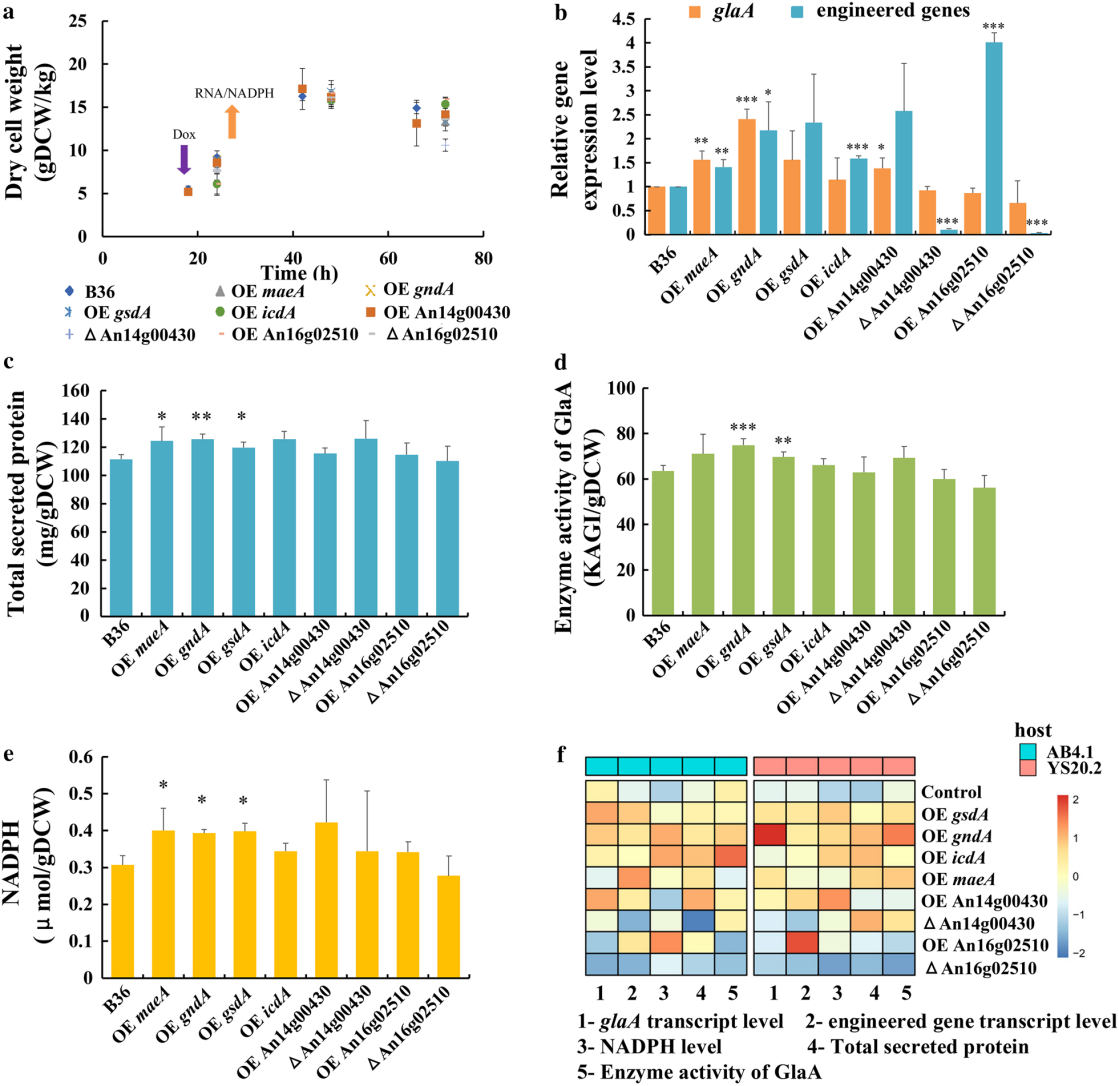
Data for shake flask-level cultivations of all engineered strains in the YS20.2 background in relation to the control strain B36. **a** Dry cell weight (DCW); **b** Relative expression level of *glaA* and engineered genes; **c** Total secreted protein per gram biomass at 72 h after inoculation; **d** Enzyme activity of GlaA per gram biomass at 72 h after inoculation; **e** Intracellular NADPH concentration in the exponential phase; **f** Comparison between engineered strains in the AB4.1 and YS20.2 background, respectively. All experiments were conducted in biological quadruplicates. Significance values were calculated with the two-tailed t-test with independent variables (*p < 0.05, **p < 0.01, ***p < 0.001)

All 14 strains were subjected to batch cultivations in shake-flask format, whereby a medium containing maltose as GlaA-inducing carbon source was used. FW35.1 (a *pyrG* + derivative of AB4.1) and B36 were taken along as corresponding reference strains. DOX-induced gene expression in all seven AB4.1 derivatives was about 1.5–2.7 times higher compared to the reference strain FW35.1 as examined by qRT-PCR (Additional file 1: Fig. S8, Table S4). Although this led to an elevated NADPH pool of about 30% in the case of *gndA*, *icdA* or An16g02510, no significant increase in GlaA enzyme activity was observed for all of seven strains compared to the FW35.1 reference (Additional file 1: Fig. S8, Table S4). However, when all seven candidate genes were overexpressed in the YS20.2 background strain containing seven *glaA* gene copies, increased transcript levels were similar as in AB4.1, but for An16g02510 higher transcription levels (fourfold) were observed (Fig. 2b). Noteworthy, overexpression of *gndA* displayed the highest effect on the transcription of *glaA* (2.4-fold) compared to B36. A NADPH pool increase of about 30% was measured in three other strains overexpressing *maeA*, *gndA*, *gsdA*, respectively (Fig. 2e). With regard to Tet-on driven gene expression of *maeA*, *gndA*, *gsdA*, about 10% increase in secreted total protein, and about 10–18% higher GlaA activity was observed for these engineered strains (Fig. 2c, d, Additional file 1: Table S4). These observations encouraged our hypothesis that NADPH engineering might be a promising strategy to improve GlaA production.They furthermore implied that the success of such an approach is dependent on the *glaA* gene copy number as the impact on protein production is not linearly correlated with increased transcription of NADPH producing enzymes. To shed further light on these phenomena, we selected the three most promising YS20.2 background strains overexpressing *maeA*, *gndA*, *gsdA*, respectively, cultivated them under chemostat conditions, and compared their performance with the reference strain B36.

### Physiology and gene expression during maltose-limited chemostat cultivations

**Fig. 3:**
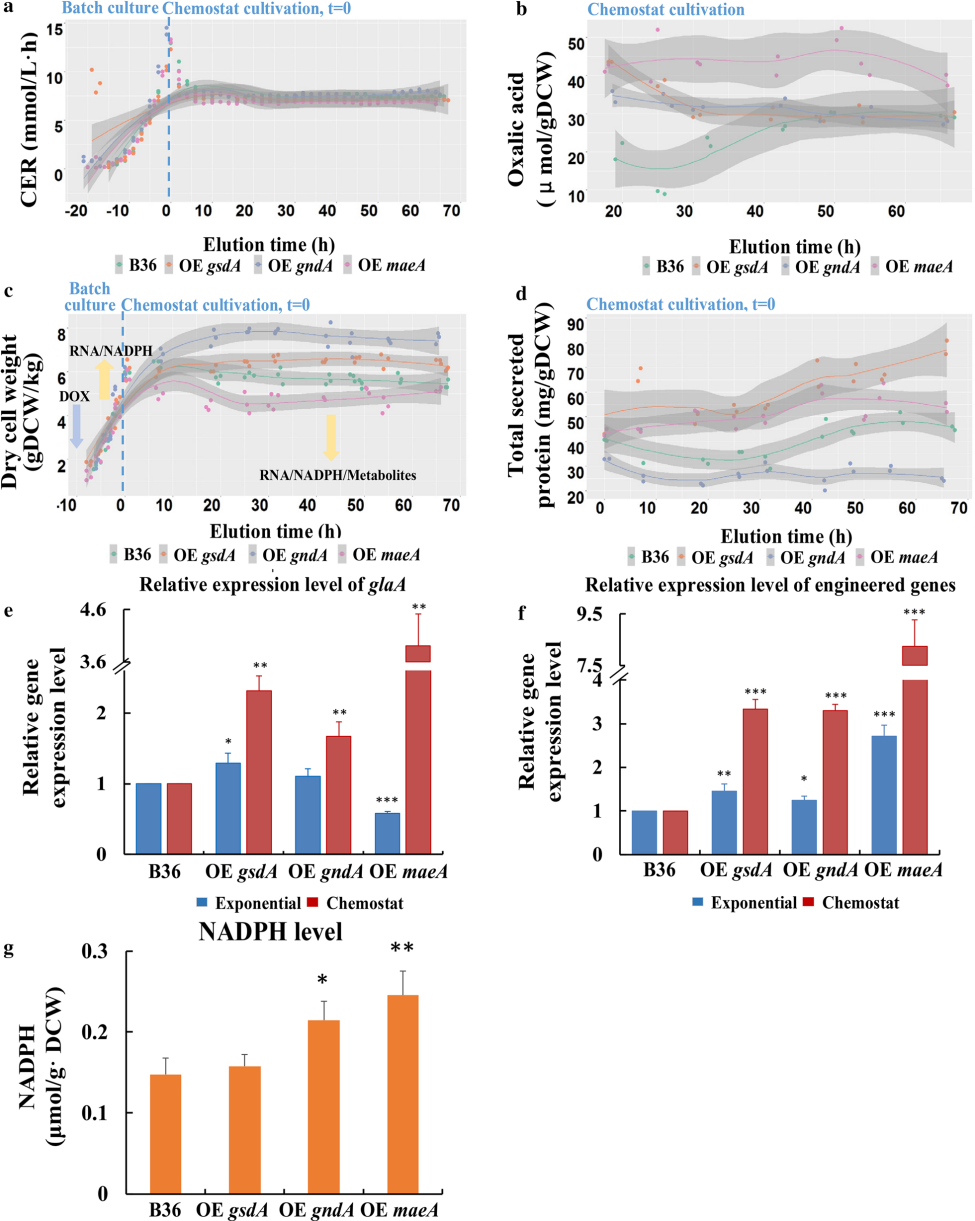
Physiological parameters for *A. niger* B36 and three overexpression strains during chemostat cultivation at a dilution rate of 0.1 h^−1^. **a** CO_2_ evolution rate (CER) in mmol/L/h; **b** Formation of the by-product oxalic acid in µmol/g cell dry weight; **c** Dry cell weight in gDCW/kg; **d** Yield of total secreted protein per gram biomass; **e** *glaA* relative gene expression level at the exponential growth phase and during steady state; **f** Relative gene expression level of the overexpressed genes during exponential growth and steady state; **g** Intracellular NADPH level at steady state. Data represent the mean ± SD from two independent cultures which were measured in technical triplicates. Significance values were calculated with the two-tailed *t*-test with independent variables (*p < 0.05, **p < 0.01, ***p < 0.001). Straight lines in a-d indicate the switch from batch to chemostat cultivation. OE *gsdA*: strain overexpressing gene *gsdA*; OE *gndA*: strain overexpressing gene *gndA*; OE *maeA*: strain overexpressing gene *maeA*

Strain B36 and the three strains overexpressing *maeA*, *gndA,* and *gsdA*, respectively, were run in duplicate maltose-limited chemostat cultures. To induce the expression of these three candidate genes, 10 µg/ml DOX was added during the early exponential growth phase, when the biomass reached 1–2 g_DCW_/kg. After about 22 h, the cultivation process was switched to the chemostat mode with a dilution rate D = 0.1 h^−1^, as described in Kwon et al. [32] and the Materials and Methods section. DOX was continuously added through the feed medium. During exponential and steady state conditions, culture samples were taken using an in-house developed fast-quenching sampling device (unpublished) for biomass determination, gene expression analyses (qRT-PCR), intracellular metabolite quantification (GC/LC–MS) and secreted protein determination. Carbon was accounted for in carbon balances of the feed medium, effluent culture broth, and exhaust gas. The carbon dioxide evolution rate (CER), the oxygen uptake rate (OUR), and biomass concentrations reached constant levels after about three-volume changes (Fig. 3a, c).

Interestingly, overexpression of *gsdA* or *gndA* increased biomass accumulation, whereas overexpression of *maeA* reduced it. This is also reflected by the final carbon-recoveries (Table 2). They were higher in both *gsdA* or *gndA* overexpressing strains (110%, 104%), but lower in *maeA* overexpressing strain (91%) compared to 99% of the B36 strain. Similar to shake flask-level cultivations, transcript levels of all three overexpressed genes were about 1.3 (*gsdA*, *gndA*) or 2.7 (*maeA*) times above their respective transcript levels in B36 during the exponential phase. They considerably increased 3.3-fold (*gsdA*, *gndA*) or 8.2-fold (*maeA*) during steady state conditions (Fig. 3e, f). This data might suggest that although all three genes are under the same Tet-on driven transcriptional control at the same locus (*pyrG*), other regulatory mechanisms, e.g., mRNA turnover or metabolic feedback regulation, might additionally control the activity of these three genes. Notably, overexpression of *gsdA* increased biomass accumulation (Table 2) but inhibited the yield of total secreted protein and GlaA by 40% (Fig. 3d), suggesting a competition between growth and protein production as previously proposed [11]. In contrast, overexpression of *gndA* and *maeA* positively impacted protein secretion by 60% and 30%, respectively (Table 2). This data was consistent with NADPH pool measurements during steady state conditions: Overexpression of *gndA* and *maeA* increased NADPH levels (46% and 66%, respectively), whereas only wild-type NADPH levels were observed for the strain overexpressing *gsdA* (Fig. 3g).

**Table 2:** Physiology data in chemostat cultures

	B36	OE *gsdA*	OE *gndA*	OE *maeA*
µ_exponential phase_ (h^−1^)	0.21 ± 0.01	0.21 ± 0.02	0.21 ± 0.01	0.19 ± 0.01
C_biomass_ (g_DCW_/kg)	5.66 ± 0.16	7.5 ± 0.36	6.46 ± 0.17	4.74 ± 0.29
q_CO2_ (mmol/g_DCW_·h)	1.33 ± 0.04	1.00 ± 0.03	1.1 ± 0.03	1.45 ± 0.09
q_O2_ (mmol/g_DCW_·h)	1.6 ± 0.18	1.19 ± 0.15	1.51 ± 0.11	1.79 ± 0.20
RQ	0.83 ± 0.09	0.81 ± 0.1	0.73 ± 0.06	0.81 ± 0.09
q_Protein_ (mg/g_DCW_·h)	3.71 ± 0.41	2.27 ± 0.37	5.62 ± 1	4.94 ± 0.49
q_s_ (mmol_maltose_ /g_DCW_·h)	0.39 ± 0.011	0.31 ± 0.009	0.35 ± 0.031	0.47 ± 0.011
Y_GlaA/X_ (U/g_DCW_)	23.84 ± 1.08	14.38 ± 0.82	38.88 ± 3.6	30.83 ± 0.1
C-recovery	99%	110%	104%	91%

Data for strain B36 and three Tet-on driven overexpression strains derived thereof are shown for chemostat cultures with maltose as growth-limiting substrate. Standard deviations ( ±) are given for mean values of duplicate independent steady state results which were measured in technical triplicates. OE *gsdA*: strain overexpressing gene *gsdA*; OE *gndA*: strain overexpressing gene *gndA*; OE *maeA*: strain overexpressing gene *maeA.* C_biomass_, biomass concentration (dry cell weight (DCW)); q_CO2_, specific carbon dioxide evolution rate; q_O2_, specific oxygen uptake rate; RQ, respiratory quotient calculated as the ratio of CO_2_ production and O_2_ consumption rates; q_protein_, specific production rate of extracellular protein; q_s_, specific substrate consumption rate; Y_GlaA/X_, yield of total glucoamylase activity on biomass; C-recovery, carbon recovery

### Metabolic differences revealed by multivariate statistical analysis

**Fig. 4:**
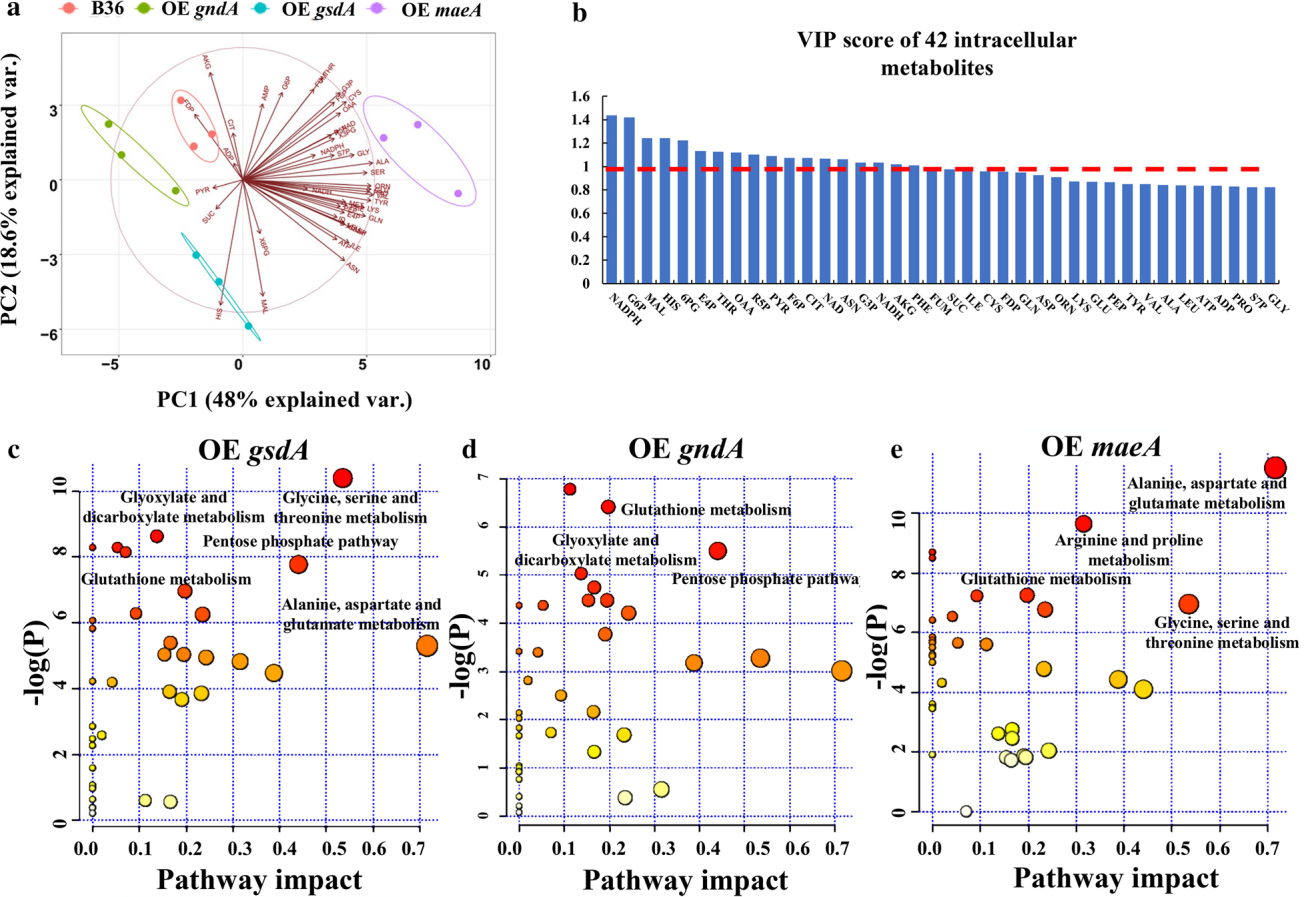
Bioplot (combining the loading plot and score plot). Three biological replicates from each strain were denoted with the same color (**a**), VIP score of 42 intracellular metabolites calculated using the PLS-DA (**b**), and respective pathway impact analysis for metabolic profiling at steady state (**c**–**e**)

In total, 42 intracellular metabolites were identified and quantified for all eight chemostat runs. These included nine sugar phosphates, eight organic acids, 19 amino acids, and six currency metabolites (NAD, NADH, NADP, NADPH, ADP, ATP). Principal Component Analysis (PCA) uncovered that samples from all four strains separated into four distinct groups as shown in the score plot of Partial Least Squares Discrimination Analysis (PLS-DA) (Fig. 4a). Especially the strain overexpressing *maeA* displayed the strongest metabolic changes, whereas the strains overexpressing *gsdA* and *gndA*, respectively, showed only subtle differences when compared to the metabolic profile of strain B36. The loading map uncovered representative metabolites which mainly contributed to the separation of these four strains. Variations of relative abundances of pyruvate (PYR), succinate (SUC), histidine (HIS), maltose (MAL), 6-phosphogluconate (6PG) mainly contributed to distinguish both *gsdA* and *gndA* overexpressing strains, whereas the majority of variables contributed to the separation of OE *maeA* strain (Fig. 4a). A variable importance plot (VIP) displaying the relative contributions of these representative metabolites demonstrated that VIP values of 18 metabolites out of the 42 metabolites were above 1 (Fig. 4b), suggesting that these 18 metabolites could be considered as potential markers to discriminate all four strains. Pathway enrichment analyses highlighted that the PPP, the glyoxylate bypass, and dicarboxylate metabolism had a significant impact in the strains overexpressing *gsdA* and *gndA*, respectively. However, glycine, serine, and threonine metabolism was enriched in the strains overexpressing *gsdA* and *maeA*. The latter in general showed overrepresentation of amino acid metabolic pathways, including alanine, aspartate and glutamate metabolism, arginine and proline metabolism besides glycine, serine and threonine metabolism (Fig. 4c–e).

### Metabolic profiling of amino acid pools and central carbon metabolism

**Fig. 5:**
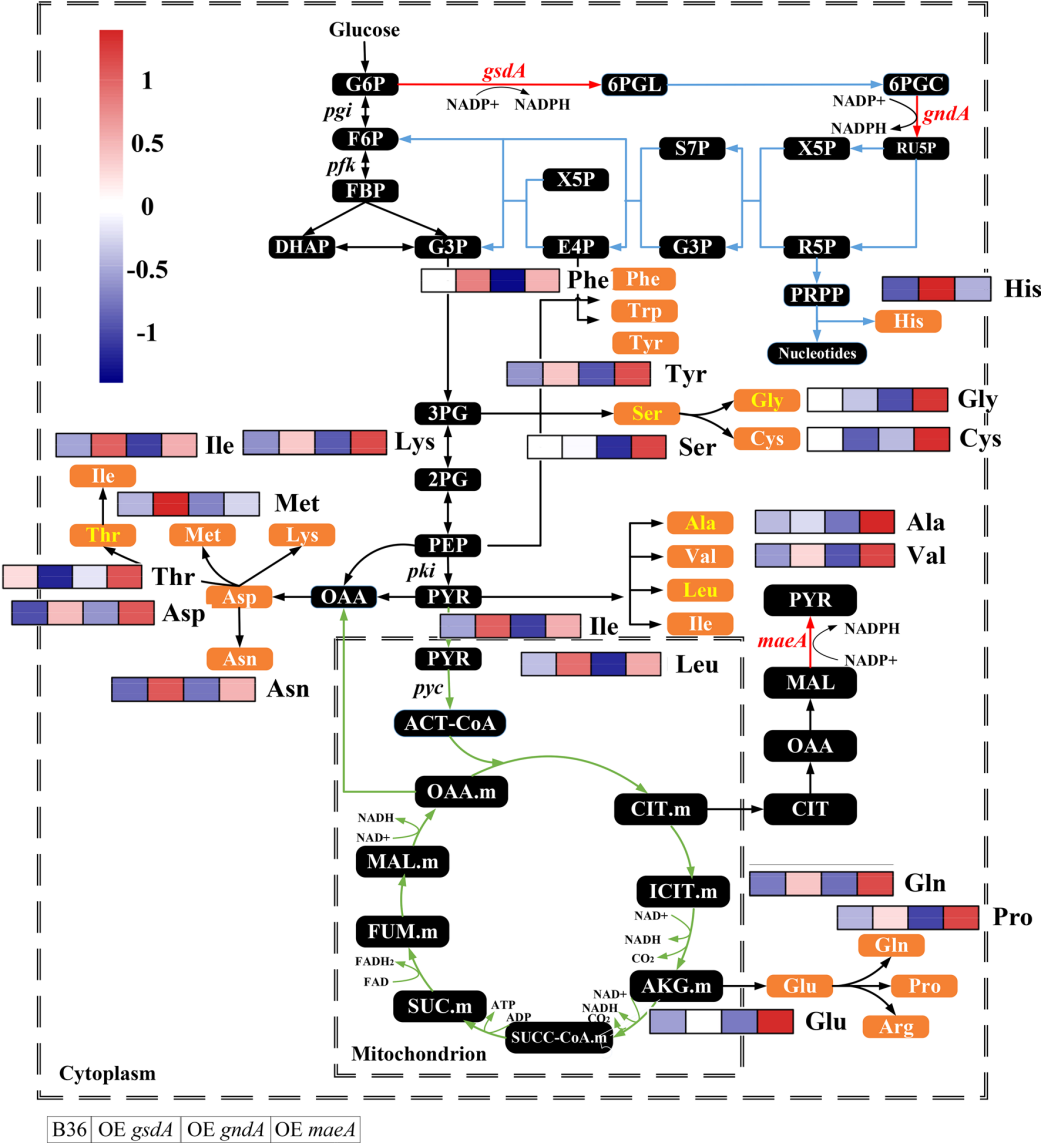
The pool sizes of amino acids for the *A. niger* reference strain B36 and three overexpression strains at steady state. Blocks in the heat map represent B36, OE *gsdA* (strain overexpressing gene *gsdA*), OE *gndA* (strain overexpressing gene *gndA*), and OE *maeA* (strain overexpressing gene *maeA*) from left to right. Amino acids labeled in yellow are the top five amino acids present in GlaA. Red arrows indicate the enzymes overexpressed. Quantified amino acid amounts and significance values are given in Additional file 1: Table S5

Figure 5 and Additional file 1: Table S5 summarize the amino acid pools in all three overexpression strains compared to B36. In general, Ala, Glu, Gly, Leu, and Lys are the top five amino acids in the biomass of *A. niger*, whereas Ser, Thr, Ala, Leu, and Gly account for about 50% of the total amino acids in GlaA [40]. Our metabolomics analyses uncovered that in all four strains, amino acids from the glutamate family are most and aromatic amino acids are least abundant, which is in general agreement to previously reported amino acid pools in *A. niger* [41]. Overall, the amino acid pool in the *gndA* overexpression strain was slightly reduced compared to B36 but was increased in strains overexpressing *gsdA* (22%) or *maeA* (30%), respectively (Additional file 1: Table S5). A general observation was also that the histidine pool dramatically increased when *gsdA* (increased by three folds) or *gndA* (60%) were overexpressed. Two out of the five amino acids dominating *A. niger*'s biomass (Glu, Lys) accumulated in the strain overexpressing *gsdA*, while the pool sizes of three top GlaA composing amino acids (Ser, Thr, Gly) were less abundant compared to B36. This data might explain the reduced GlaA production in this strain. In the case of *maeA* overexpression, nearly all amino acids where higher abundant when compared to strain B36 (Additional file 1: Table S5), especially the pools for the GlaA dominating amino acids Thr, Ala, Leu, and Val, suggesting that increased amino acid pools might cause the extra driving force for GlaA formation in a *maeA* overexpressing strain. In contrast, the overall amino acid pools of the strain overexpressing *gndA* displayed only moderate reduction compared to B36 (Additional file 1: Table S5).

**Fig. 6:**
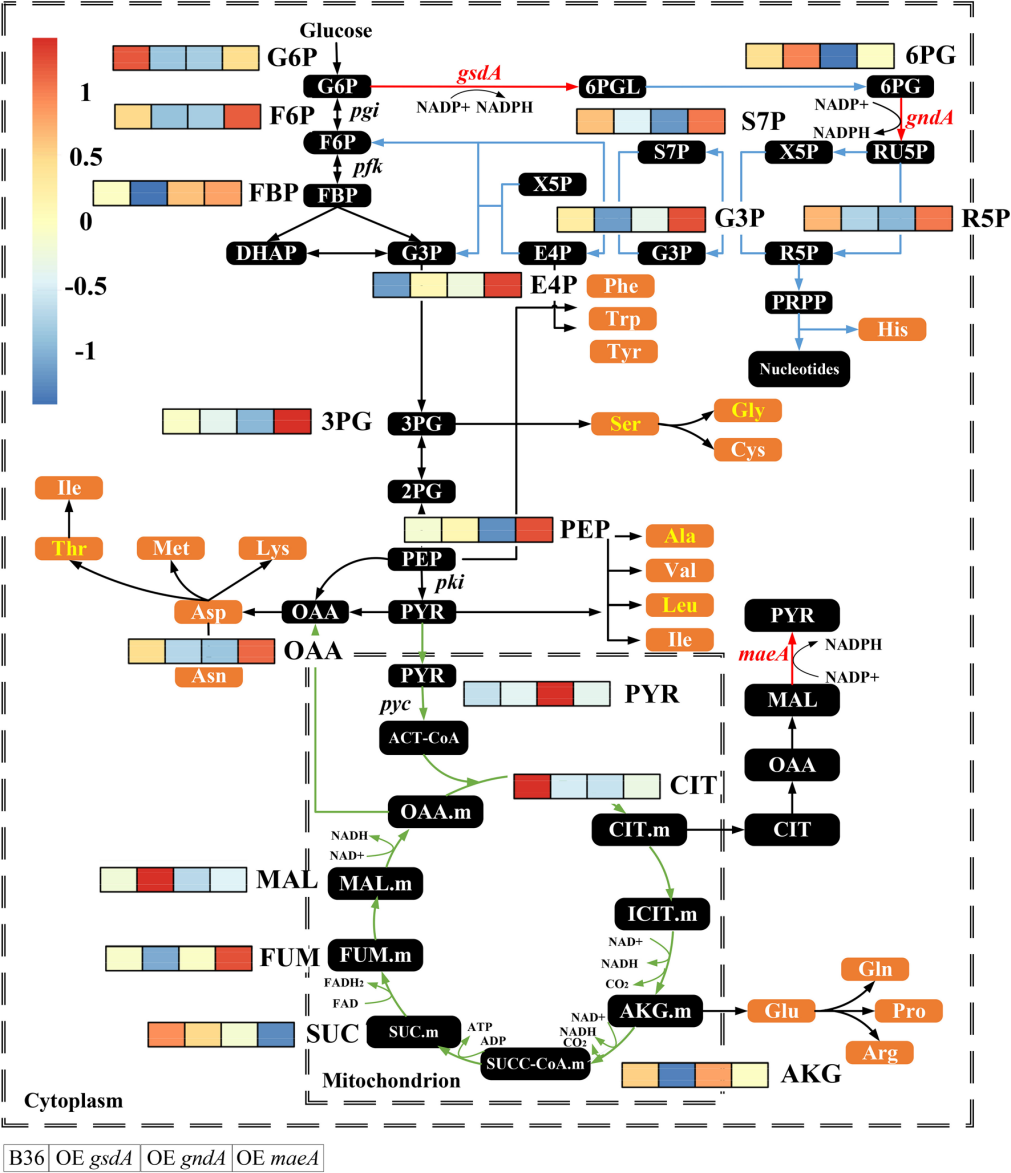
The pool sizes of a part of the metabolites on central carbon metabolism pathways for the *A. niger* reference strain B36 and three engineered strains at steady state. Blocks in the heat map represent B36, OE *gsdA* (strain overexpressing gene *gsdA*), OE *gndA* (strain overexpressing gene *gndA*), and OE *maeA* (strain overexpressing gene *maeA*) from left to right. Red arrows indicate the enzymes overexpressed. Quantified sugar and organic acid amounts including significance values are given in Additional file 1: Table S6

As summarized in Fig. 6 and Additional file 1: Table S6, overexpression of *gsdA*, *gndA,* and *maeA*, led to a significant flux redistribution of the central carbon metabolism, including PPP, EMP, and TCA. As expected, the glycolytic pathway intermediates G6P and F6P were significantly reduced in the two PPP engineered strains which overexpressed *gsdA* and *gndA*, respectively. Diverting the carbon flux towards the PPP at the branch node of G6P after overexpression of *gsdA* and *gndA* has in fact lowered the flux towards the EMP. The 6PG and PEP pools increased (due to *gsdA* overexpression), which in turn inhibited the upper glycolytic pathway (feedback inhibition via 6PG [42] and PEP [43]). Another consequence is a reduced carbon uptaken rate, which is indeed the case for the strain overexpressing *gsdA* (Table 2). On the lower glycolytic pathway, the accumulation of the intermediates PEP and PYR, respectively, in OE *gsdA* and OE *gndA* led to a reduced flux through 3PG (Additional file 1: Fig. S9), accompanied by a reduction of the serine family pool in these two strains. This data could be well explained by the close association between the abundance of metabolite precursors on the central metabolic network and the pool size of the correlated amino acid families as already reported elsewhere [44]. Importantly, the PPP is not the only key for NADPH regeneration but also provides two important precursors for amino acid biosynthesis, i.e., ribose 5-phosphate (R5P) and erythrose 4-phosphate (E4P), which in turn are required for His and aromatic amino acid biosynthesis. Overproduction of *gndA* channeled more carbon from 6PG to Ru5P, resulting in a lower abundance of 6PG as expected. Surprisingly, the R5P pools declined in both strains overexpressing *gsdA* and *gndA*, respectively. However, taking into account the increased biomass formation and higher His pools in these two strains, the increased carbon flux towards R5P was possibly channeled towards nucleotide biosynthesis. In agreement with previous reports studying high protein secretion in *A. niger* [18, 41] and our in silico metabolic flux simulations (Additional file 1: Fig. S9), the flux through the TCA cycle was also reduced during overexpression of the PPP genes *gsdA* and *gndA*, respectively. However, the picture for the TCA cycle intermediates was inhomogeneous. The TCA cycle intermediates AKG, FUM, and OAA were less abundant, whereas MAL accumulated when *gsdA* was overexpressed. In the case for *gndA* overexpression, the experimental data showed only weak differences for the pool sizes of TCA intermediates when compared to strain B36 (Fig. 6, Additional file 1: Fig. S9). Last but not least, overexpression of *maeA* facilitated not only amino acid biosynthesis but also improved the carbon flux towards the EMP and TCA cycle (Fig. 6) and elevated the pools of the key intermediates F6P, G3P, PEP, OAA, and FUM but not PYR. Worth mentioning in this context is oxalic acid, a major by-product during *A. niger* cultivation which stems from OAA [45]. Whereas the oxalic acid pool did not differ in the strains overexpressing *gsdA* or *gndA*, respectively, it was substantially increased by about 30% in OE *maeA,* suggesting that the elevated pool size of OAA could be responsible for the accumulation of this by-product (Fig. 3b).

## Discussion

NADPH regeneration is a limiting step for amino acid biosynthesis; hence NADPH availability and allocation are essential for efficient protein production. This study demonstrates that the NADPH pool can be increased by 30% in *A. niger* through genetic engineering by the *gndA*, *gsdA,* and *maeA* genes, respectively. However, the physiological consequences of this increased NADPH pool are strain-dependent. For the native GlaA producing strain AB4.1, which carries only one *glaA* copy, we assume that the excess of NADPH might not be allocated to increased amino acid biosynthesis but was channeled to other NADPH consumption pathways, e.g., for the production of steroids, lipids, and nucleotides and thus biomass formation. In contrast, due to the higher *glaA* gene number in B36, it is tempting to speculate that the excess in NADPH was indeed used for GlaA production because of the higher intracellular pull towards GlaA production and its amino acid precursors. Future ^13^C metabolic flux analyses targeting sugars, amino acids, steroids, lipids, and nucleotides in these strains will prove or disprove this hypothesis. These analyses will also clarify whether NADPH supply is a bottleneck for protein biosynthesis, as previously proposed for the yeast *Y. lipolytica* [46].

Metabolic flux analysis is the most authoritative method for measuring in vivo fluxes [47]. In this study, we used our previously updated *A. niger* GSMM iHL1210 [31] to predict the flux distribution of the central carbon metabolic network in silico during steady-state conditions of three strains overexpressing *gndA*, *gsdA,* and *maeA*, respectively. These simulation results indicated that the relative flux of the EMP and TCA cycle decreased after redirecting a higher flux through the PPP when *gndA* or *gsdA* were overexpressed, a situation which was reverse predicted when *maeA* was overexpressed. This predicted computational flux distribution was congruent with the in vivo metabolite pools measured by mass spectrometry: The upper glycolytic pathway intermediates G6P and F6P were significantly lower (*p* < 0.05) in the two strains overexpressing *gndA* or *gsdA*, which both encode enzymes of the PPP. When compared with other autonomously evolved metabolic networks from forced protein overproduction [18, 41, 48], it illustrates a comparable flux pattern through the PPP and TCA cycle as observed here, suggesting similarities of core carbon metabolism in the central metabolic network in diverse high-yield protein overproducing *A. niger* cell factories.

Notably, overexpression of the *gsdA* gene improved biomass accumulation and only slightly increased NADPH production, which were paralleled by reduced protein production capacity by about 40%. This observation is generally consistent with previous reports for *A. niger* and other filamentous fungal cell factories. For example, a high specific protein production rate has been shown to correlate with relatively low growth rates in *Trichoderma reesei* [49], due to a reduced proteome allocated for central metabolism [12]. Also, a recent multi-omics analysis from our group that integrated transcriptomics, metabolomics and GSMM simulations proposed that an increased GlaA production is probably achieved through reduced growth, which likely is regulated at several metabolic mechanisms: (i) an increased carbon catabolism that generates more amino acid precursors for protein production, (ii) a reduced fatty acid and ribosome biogenesis and, thus, reduced growth, and (iii) an increased flux through the glyoxylate bypass to reduce NADH formation from the citric acid cycle and to maintain the cellular redox balance [50]. In the current study, at least for the two strains overexpressing *gndA* and *gsdA*, respectively, we observed that both the PPP and the glyoxylate pathway changed considerably (Fig. 4). The general view might be that once growth is limited, more reducing equivalents (NADH and NADPH), and precursors can be channeled into protein and glucoamylase production. We have shown previously that *A. niger* benefits from a very flexible transcriptional machinery that ensures adaptation to the burden of high protein loads [32]. When forced to overexpress *glaA*, *A. niger* increases the transcription of secretory pathway genes involved in translation, protein folding, and protein secretion. Under this circumstance, the expression of genes less important for growth and survival becomes decreased loads [32]. This phenomenon is called 'Repression under secretion stress' (RESS) and was first discovered in T*. reesei* [51]. *A. niger* can, thus, fall back on a very efficient gene regulatory and metabolic machinery that balances cellular capacities with the intracellular burden. However, it is currently impossible to predict nor to understand all regulatory mechanisms and their correlations behind these phenomena. Additionally, ^13^C-flux analysis detected that the two NADPH generating reactions, i.e., NADP-ICDH and G6PDH, contributed only negligibly (below 5%) to the total NADPH production [52]. This could be an alternative reason for the minor changes in NADPH when comparing the strain overexpressing *gsdA* to the reference strain B36. Moreover, more NADPH might have been consumed for growth in this strain. Yet, this observation is opposite to the critical role of G6PDH in the production of NADPH in *Saccharomyces cerevisiae* [53]. Our study clearly uncovered that overexpression of the *gndA* or *maeA* gene, respectively, enhanced total protein production and specifically GlaA production in *A. niger*. Whereas the *gndA* gene encodes one enzyme of the PPP, the *maeA* encoded enzyme is part of the cytosolic reverse TCA cycle. Overexpression of both genes gave rise to both the NADPH pool (46%, 66%, respectively) and protein production rates (65%, 30%, respectively) compared to B36, thus implying that our in silico predicted cofactor engineering approach is a biological valid and thus successful approach to improve protein production capacities of *A. niger*. Notably, overexpression of *maeA* provoked the most complex metabolic changes (Fig. 5 and 6). NADP-ME is critical not only for pyruvate supply but also enhanced the flux through glycolysis and TCA cycle to provide sufficient precursors for amino acid production. Based on our previous multi-omics analysis and exogenous amino acid addition experiments [50], Ala, Glu, Gly, and Asp were confirmed as four amino acid limiting GlaA production in *A. niger*. This was further corroborated by our recent study reporting that removing the amino acid limitation is supporting GlaA production [54]. Hence, enriched pools of amino acids, carbon precursors and NADPH due to increased *maeA* transcription could altogether boost GlaA production. However, increased *maeA* transcription was paralleled by the accumulation of the by-product oxalic acid, which could in turn be targeted in the next DBTL cycle to even further increase GlaA production.

## Conclusions

In this study, we followed a DBTL approach to systematically understand and optimize GlaA production in *A. niger*. Our previous multi-omics integration analyses predicted that NADPH regeneration could be one so far unstudied bottleneck for enzyme production in *A. niger*. To test this hypothesis, we genetically engineered strains with improved NADPH biosynthetic capacities. Metabolic profiling and multivariate statistical analysis revealed that three NADPH regeneration enzymes encoded by the *gsdA*, *gndA,* and *maeA* genes play the most significant decisive role during batch and continuous cultivations, whereby overexpression of the *gndA* and *maeA* genes provoked the highest flux redistributions towards protein biosynthesis. Future studies will examine whether combined overexpression of these candidate genes, e.g., by polycistronic gene expression [55], will further improve protein production capacities of *A. niger* and thus evaluate whether combinatorial cofactor engineering is a further successful approach for strain engineering.

## Materials and methods

### Plasmids construction

Integrative plasmids used for overexpressing NADPH generating genes in the *A. niger* strains were cloned by Gibson assembly or AQUA cloning [56]. The backbone vector was obtained by digesting pFW22.1 with *Pme*I, and then the 8.1 kb fragment was purified by gel extraction. Plasmid pFW22.1 carries the Tet-on inducible gene expression system, and an unfunctional *pyrG*, which was used as a selective marker for transformant screening [35]. Open reading frames (ORF) of NADPH generating enzymes (6PGDH (*gsdA*), G6PDG (*gndA*), NADP-ICDH (*icdA*), NADP-ME (*maeA*), An14g00430, An15g04590, and An16g02510) were amplified by PCR, using genomic DNA from the *A. niger* B36 strain as a template. PCR products were integrated into pFW22.1 at the *Pme*I site (Additional file 1: Fig. S2). Primers carrying overlapping regions to the backbone vector and plasmids constructed in this study are listed in Additional file 1: Tables S7 and S8, respectively.

### Strains

*A. niger* strains used in this study are listed in Table 3. AB4.1 is a lab strain with only one *glaA* copy and uridine auxotrophic [36]. B36 is an engineered strain with multiple copies of *glaA* and derived from N402 [37]. YS20.2 is a uridine auxotrophic derivative of B36, which was engineered using CRISPR/Cas9 (see below). Overexpression strains of NADPH generating genes were constructed in the background of AB4.1 or YS20.2, and transformants were screened based on uridine prototrophy. Gene deletions were obtained using the split marker method [57]. PCR products containing either ~ 1.5 kb 5′ or 3′ flanking regions of the corresponding gene and a part of the selective marker hygromycin were used for transformation (for details please refer to Additional file 1: Fig. S4 and Table S7). To avoid a lethal phenotype, all deletion strains were built in their corresponding overexpression strains. *A. niger* transformation, genomic DNA extraction, and Southern hybridization were performed as previously described in Arentshorstet et al. [58] with the following exceptions: strain YS20.2 was cultivated at 80 rpm to obtain young mycelium for protoplastation. PEG mediated transformation was done using 60% PEG 4000 as described in Pohl et al. [38]. This improved the transformation rate from 1 CFU/µgDNA to 4–5 CFU/µgDNA. All overexpression and deletion strains were generated without CRISPR. Positive transformants were confirmed by diagnostic PCR and Southern analysis (Fig. S3, S5, S6, and S7).

**Table 3:** *Aspergillus niger* strains used in this study

Strain name	Background strain	Relevant genotype/description	References
AB4.1	N402	*cspA1*-, *pyrG* −	[36]
FW35.1	AB4.1	*cspA1*-, *pyrG* +	[35]
B36	N402	Multi copies of *glaA*, *amdS* +	[37]
YS20.2	B36	*pyrG*-, with 195 bp deletion at 101 bp ~ 295 bp after *pyrG* start codon	This study
YS7.4	AB4.1	Overexpression of An02g12140 (*gsdA*) via Tet-on, *pyrG* +	This study
YS23.20	YS20.2	Overexpression of An02g12140 (*gsdA*) via Tet-on, *pyrG* +	This study
YS9.9	AB4.1	Overexpression of An11g02040 (*gndA*) via Tet-on, *pyrG* +	This study
YS22.17	YS20.2	Overexpression of An11g02040 (*gndA*) via Tet-on, *pyrG* +	This study
YS10.6	AB4.1	Overexpression of An02g12430 (*icdA*) via Tet-on, *pyrG* +	This study
YS37.10	YS20.2	Overexpression of An02g12430 (*icdA*) via Tet-on, *pyrG* +	This study
YS12.16	AB4.1	Overexpression of An05g00930 (*maeA*) via Tet-on, *pyrG* +	This study
YS21.14	YS20.2	Overexpression of An05g00930 (*maeA*) via Tet-on, *pyrG* +	This study
YS11.8	AB4.1	Overexpression of An14g00430 via Tet-on, *pyrG* + Overexpression of An14g00430 via Tet-on, *pyrG* +	This study
YS24.9	YS20.2		
YS16.1	YS11.8	An14g00430::*hygB*	This study
YS26.3	YS24.9	An14g00430::*hygB*	This study
YS14.4	AB4.1	Overexpression of An16g02510 via Tet-on, *pyrG* +	This study
YS38.2	YS20.2	Overexpression of An16g02510 via Tet-on, *pyrG* +	This study
YS15.7	YS14.4	An16g02510::*hygB*	This study
YS35.9	YS38.2	An16g02510::*hygB*	This study

### Medium

Strains were grown at 30 °C using a complete or minimal medium supplemented with 1 mM uridine when necessary [58]. Shake flask medium (g/L): 3% Maltose·H_2_O, 10 g tryptone, 5 g Yeast extract, 1 g KH_2_PO_4_, 0.5 g MgSO_4_·7H_2_O, 0.03 g ZnCl_2_, 0.02 g CaCl_2_, 0.0076 g MnSO_4_·H_2_O, 0.3 g FeSO_4_·7H_2_O, 3 ml Tween 80, pH was adjusted to 5.5 by 1 M HCl. Maltose-limited chemostat medium was as described in Kwon et al. [32] with the following slight modification: the medium contained 1% (w/v) of maltose in batch cultures and 0.8% (w/v) during chemostat cultivations. Germination was induced by the addition of 0.003% (w/w) yeast extract. Media used for CRISPR/Cas9 gene editing: 200 µg/ml hygromycin and 500 µg/ml caffeine were supplied into MM transformation media. Filtered-sterilized 0.75 g/L 5-FOA, 10 mM proline and 10 mM uridine were added to MM medium when subcultivating uridine auxotrophic transformants.

### CRISPR/Cas9 genetic modification

CRISPR/Cas9 modifications were done using the ribonucleoprotein (RNP) approach [38]. The Cas9 protein was expressed from the pET28aCas9cys vector (Addgene: 53,261) and purified by AKTA FPLC (Fast protein liquid chromatography, GE, USA). The sgRNA was selected online using the Cas-Designer website (https://www.rgenome.net/cas-designer/). Two sgRNAs were designed to test the gene targeting efficiency, which located at 205 bp and 393 bp after the start codon of *pyrG*. DNA templates for in vitro sgRNA synthesis (MegaScript T7 Transcription Kit, Thermo Fisher Scientific, USA) were constructed as DNA oligos incorporating a T7-promoter sequence, 20 bp protospacer and a 77 bp sgRNA tail (Primers are listed in Additional file 1: Table S9). During transformation, 5 µl purified Cas9 protein, 1 µl or 2 µl sgRNA, and 2 µg pMA171.1 plasmid carrying hygromycin as a selective marker [59] were used. Transformants were subcultivated on MM plates containing uridine, proline, and 5-FOA (details on concentrations are above). Consequently, the ORF region of *pyrG* from sporulating single colonies on 5-FOA medium was amplified to confirm mutations by sequencing (Additional file 1: Fig. S1).

### Shake flask-level cultivation

To evaluate the performance of engineered strains as GlaA producers, 10^6^ spores/ml of reference strains FW35.1 [35] or B36 [32] and engineered strains were inoculated into 50 ml shake flask liquid medium and cultivated at 30 °C and 250 rpm. After 18 h of cultivation, 20 µg/mL doxycycline (DOX) was used to induce gene expression. In order to ensure gene overexpression, the same amount of DOX was added every 12 h after the initial induction. Samples were taken at 24, 48, and 72 h after inoculation. Physiological parameters (dry weight, total secreted protein, residual glucose, and enzyme activity of GlaA in the broth) were measured. Samples for NADPH measurement and qRT-PCR were taken in the exponential phase. Experiments were performed in biological quadruplicates.

### Chemostat cultivation

Submerged cultivations were carried out in 5 L bioreactors (NCBIO, Shanghai, China). Batch cultivations were adapted from Kwon et al. [32]. Chemostat cultivations were initiated in the late exponential growth phase when OUR (Oxygen Uptake Rate) or CER (Carbon-dioxide Evolution Rate) started to decrease and DO (Dissolved Oxygen) started to increase. The dilution rate (D) was set at 0.1 h^−1^. The steady-state was reached after approximately three residence times (≈ 30 h) and indicated by constant CO_2_, O_2,_ and biomass concentrations. Samples were taken regularly (6–8 h) to monitor growth and to determine if a steady-state had been reached. All samples were quickly frozen in liquid nitrogen. 10 µg/ml DOX was added when the biomass reached 1–2 g/kg. To ensure induction, 10 µg/mL DOX was also applied to the feed medium. Samples used for extracting intracellular metabolites were taken during steady-state and intracellular metabolites were quantified by GC/LC–MS. Samples for NADPH measurement and qRT-PCR were taken both in the mid-exponential phase (4 h after DOX induction) and at steady-state.

### Determination of dry weight, total secreted protein, residual glucose and enzyme activity of glucoamylase

4 mL of samples were taken at the indicated time points from shake flask or bioreactor cultures. Biomass was harvested through vacuum filtration, washed 3 times with deionized water, then frozen at − 80 °C and finally freeze-dried overnight to determine dry weight. Total extracellular protein in the culture supernatant was determined via the Bradford assay (BioRad, California, USA) according to the manufacturer's protocols, and absorbance (600 nm) was measured using a GloMax®-Multi Detection System (Promega, Madison, USA). Quantification of residual glucose in the cultivation medium was performed with the Glucose GOD/PAP Liqui-color kit (Human, Wiesbaden, Germany) according to the manufacturer's manual. The enzyme activity of GlaA was measured as described in Lu et al. [50].

### Quantification of extracellular organic acid

By-products such as extracellular acetic acid, citric acid, oxalic acid, pyruvic acid, and succinic acid were determined by HPLC (Shimadzu, Kyoto, Japan) using a VARIAN Metacarb H plus column and 5 mM H_2_SO_4_ as a mobile phase at a flow rate of 0.4 ml/min and 50 °C. Acids were detected at a wavelength of 210 nm.

### Quantification of intracellular metabolites

Quantification of intracellular metabolites was based on Lu et al. [41], and slight modifications were made. This study adopted the Isotope Dilution Mass Spectrometry (IDMS) method to accurately quantify intracellular metabolites [60]. 1–2 ml broth was rapidly taken from bioreactors by fast-sampling equipment to tubes with 10 ml precooled quench solution (− 27.6 °C 60% v/v methanol solution) at steady-state. To precisely determine the amount of broth taken, the tubes were weighed before and after sampling. In order to remove the extracellular metabolites promptly, the mixture was filtered with a vacuum pump. Then, 20 ml precooled quench solution was used to rinse the filter cake. The washed filter cake and 100 µl ^13^C internal standard solution were added to prewarmed 25 ml 75% (v/v) ethanol solution and extracted 3 min at 95 °C. The tubes were cooled down on the ice to room temperature. The filtrate was collected after vacuum filtration and concentrated to 600 µl by rotary evaporation. Subsequently, the metabolite pools were quantified with the LC–MS/MS (Thermo Fisher Scientific Corporation, USA) and GC–MS (Agilent, Santa Clara, CA, USA).

### Quantification of intracellular NADPH

1 ml broth was quickly taken from the shake flask or bioreactor and frozen immediately in liquid nitrogen. Samples were thawed before measurement, diluted 2 or 5 times with 1 × PBS, and centrifuged at 12,000 rpm for 5 min to remove the supernatant. Intracellular NADPH was quantified by the EnzyFluo™ Assay Kit (BioAssay Systems Corporation, USA) according to the manufacturer's manual.

### Quantitative real-time PCR

Mycelium harvested for RNA extraction was ground in liquid nitrogen and then extracted by the Fungal Total RNA Isolation Kit (Sangon, Shanghai, China). About 1 μg of total RNA was used for cDNA synthesis using the PrimeScript™ RT reagent Kit with gDNA Eraser (Takara, Shiga, Japan) according to the manufacturer's instructions. The real-time PCR reaction system was prepared with TB Green™ *Premix Ex Taq*™ II (Takara, Shiga, Japan) in a volume of 25 μl with diluted cDNA (about 1 µg) as a template. Diluted cDNA was used to keep mean Ct (threshold cycles) values between 20 and 30. Each reaction was carried out in triplicates. Oligonucleotide primers used for qPCR are listed in Additional file 1: Table S7. The 28S rRNA and 18S rRNA were used as the internal standard. PCR conditions were as follows: 95 °C for 3 min, followed by subsequent 40 cycles of the three-steps: 95 °C for 30 s, 58 °C for 30 s and 72 °C for 30 s.

### Multivariate statistical analysis of intracellular metabolites

Hierarchy clustering analysis of metabolomics data at steady state was plotted using the R package pheatmap. Principal component analysis and partial least squares discriminant analysis (PLS-DA) were then performed by the R package ggbiplot. Pathway enrichment analysis was performed by the online metabolomics analysis website MetaboAnalyst 4.0 (https://www.metaboanalyst.ca/MetaboAnalyst/faces/home.xhtml).

### Simulation of relative metabolic flux by *A. niger* GSMM

Relative metabolic fluxes were predicted as described in Lu et al. [50] through *A. niger* GSMM iHL1210, which was updated recently by our lab [31]. Maximization of cell growth was set as the objective function, and maltose was the sole carbon source. q_S_, q_P_, q_O2_, q_CO2_ from the chemostat fermentations were set as constraints during the simulation.

## Supplementary information

**Additional file 1: Fig. S1−S8, Tables S1–S9.**

## Data Availability

All data generated or analysed during this study are included in this published article.

## Abbreviations

PPP: Pentose phosphate pathway
EMP: Embden-Meyerhof-Parnas pathway
TCA: Tricarboxylic acid cycle
CER: Carbon dioxide evolution rate
OUR: Oxygen uptake rate
DO: Dissolved oxygen
PLS-DA: Partial least squares discrimination analysis
G6PDH: Glucose 6-phosphate dehydrogenase
6PGDH: Phosphogluconate dehydrogenase
NADP-ICDH: NADP + dependent isocitrate dehydrogenase
NADP-ME: NADP + dependent malic enzyme
3PG: 3-Phosphoglycerate
3HBCoA: (S)-3-Hydroxybutanoyl-CoA
AACCoA: Acetoacetyl-CoA
AC: Acetate
ACCoA: Acetyl-CoA
ACAL: Acetaldehyde
AKG: α-Ketoglutaric acid
Ala: Alanine
Arg: Arginine
Asn: Asparagine
CIT: Citrate
DHAP: Dihydroxyacetone-phosphate
DHF: 7:8-Dihydrofolate
E4P: Erythrose-4-phosphate
ETH: Ethanol
F6P: Fructose-6-phosphate
FBP: 1,6 Fructose diphosphate
FUM: Fumarate
GLC: Glucose
G1P: Glucose-1-phosphate
G6P: Glucose-6-phosphate
GlaA: Glucoamylase
GLCNT: Gluconate
G3P: Glyceraldehyde-3-phosphate
Gln: Glutamine
Glu: Glutamate
His: Histidine
ICT: Isocitrate
Ile: Isoleucine
Leu: Leucine
Lys: Lysine
Mal: Malate
Met: Methionine
OA: Oxalate
OAA: Cytosolic oxaloacetate
Orn: Ornithine
PEP: Phosphoenolpyruvate
Phe: Phenylalanine
Pro: Proline
PYR: Pyruvate
R5P: Ribose-5-phosphate
Ru5P: Ribulose-5-phosphate
S7P: Sedoheptulose-7-phosphate
Ser: Serine
SUC: Succinate
Thr: Threonine
Trp: Tryptophan
Tyr: Tyrosine
Val: Valine
X5P: D-xylulose-5-phosphate
