# Supplementary material for: Engineering cofactor metabolism for improved protein and glucoamylase production in Aspergillus niger
Source: Microb Cell Fact. 2020 Oct 23;19:198. doi: 10.1186/s12934-020-01450-w (PMC7584080; doi:10.1186/s12934-020-01450-w)
Supplement: Supplementary file 1 — Additional file 1: Fig. S1−S8, Tables S1–S9. [file 12934_2020_1450_MOESM1_ESM.pdf]

Engineering cofactor metabolism for improved protein and glucoamylase production in *Aspergillus niger*

Yufei Sui[1, 2], Tabea Schütze[2], Liming Ouyang[1], Hongzhong Lu[3], Peng Liu[1], Xianzun Xiao[1], Jie Qi[1], Ying-Ping Zhuang[1, *] and Vera Meyer[2, *]

[1] State Key Laboratory of Bioreactor Engineering, East China University of Science and Technology, Shanghai 200237, P. R. China

[2] Chair of Applied and Molecular Microbiology, Institute of Biotechnology, Technische Universität Berlin, Straße des 17. Juni 135, 10623 Berlin, Germany

[3] Department of Biology and Biological Engineering, Chalmers University of Technology, Kemivägen 10, SE412 96 Gothenburg, Sweden

*** Correspondence:**

Vera Meyer：vera.meyer@tu-berlin.de

Yingping Zhuang：ypzhuang@ecust.edu.cn

Supplemental data

Supplemental Figure

Figure S1 3

Figure S2 5

Figure S3 6

Figure S4 9

Figure S5 10

Figure S6 12

Figure S7 14

Figure S8 15

Figure S9 17

Supplemental Table

Table S1..4

Table S2..6

Table S3..12

Table S4..16

Table S5..18

Table S6..19

Table S7..20

Table S8..22

Table S9..22

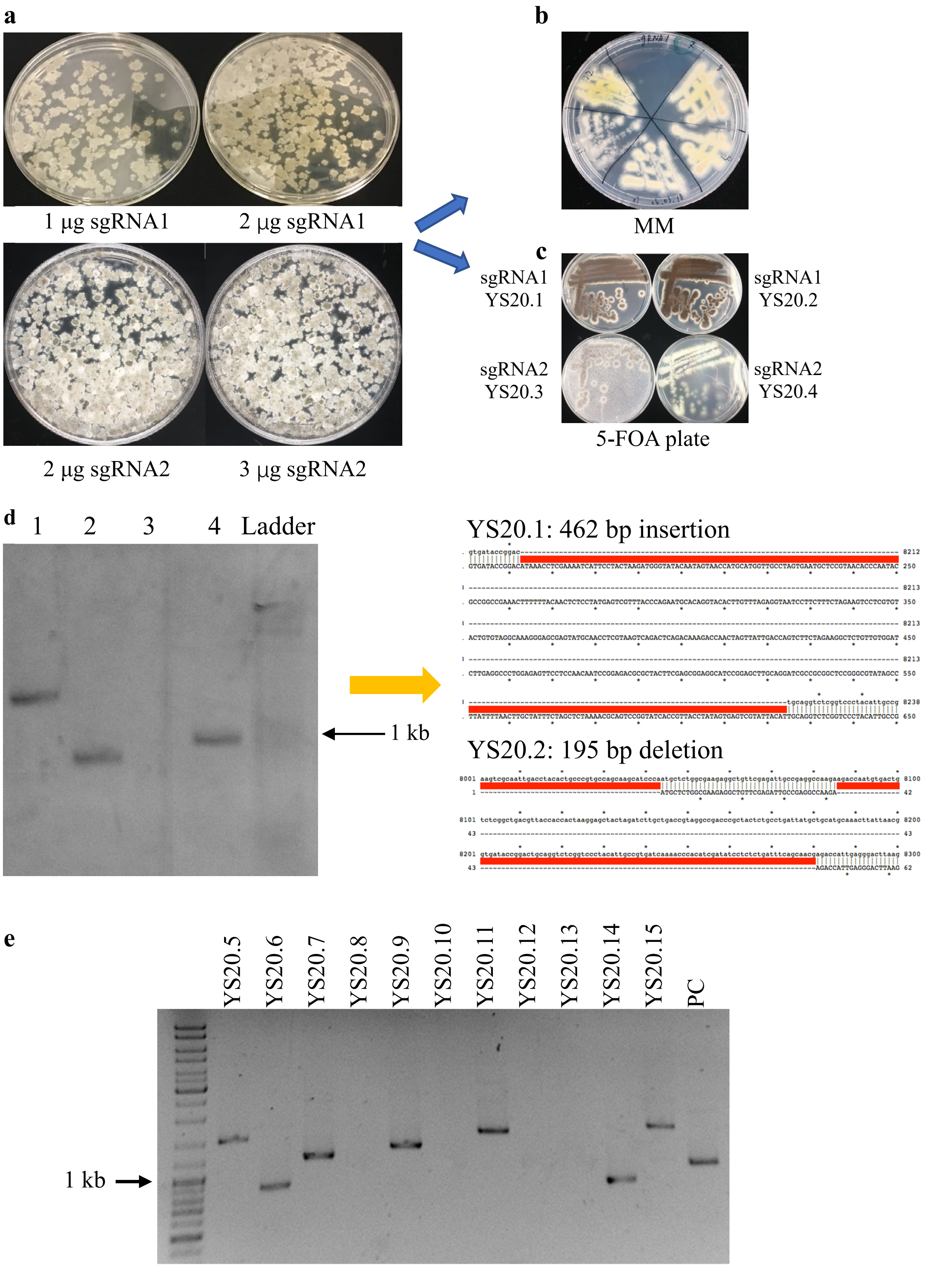

Figure S1 Generation of a *pyrG*⁻ mutant of B36. (a) Primary transformation plates supplemented with hygromycin. More colonies were obtained when 2 µl sgRNA was used compared to 1 µl, while 1 µl sgRNA is sufficient to obtain enough colonies for subsequent

screening. (b) Growth of primary transformants on MM plates. Several primary transformants were unable to grow on MM medium as expected. (c) Subcultivation of primary transformants on 5-FOA plates. Positive transformants were sporulating. (d) PCR on *pyrG* open reading frame by primer pairs 542/169 (Table S7). 1. sgRNA1 YS20.1; 2. sgRNA1 YS20.2; 3. sgRNA2 YS20.3; 4. Unmutated *pyrG*. After aligned with the wild type *pyrG* from B36, results show that YS20.1 carries a 462 bp insert (289 bp from AMA1 sequence) at 4 bp upstream of the PAM site, and YS20.2 carries a deletion of 195 bp at 101-295 bp after the start codon of *pyrG*. Both disruptions inactivated the function of *pyrG* in B36. However, no PCR product was obtained for YS20.3. (e) ORF of *pyrG* amplified from transformants YS20.5 - YS20.15. PCR results for the *pyrG* open reading frame of other transformants. The sequencing results are summarized in Table S1 and show that B36 prefers to repair genomic double-strand breaks by visible long fragment insertion or deletion. After multiple rounds of cultivation under non-selective conditions, we observed that the hygromycin resistance in all the above-mentioned B36 uridine auxotrophic mutants was lost (data not shown). Subsequently, YS20.2 with a truncated *pyrG* ORF was selected as the chassis strain for further cofactor engineering.

Table S1 Summary of B36 uridine auxotrophic mutants obtained in this study. YS20.4 was unable to sporulate on 5-FOA medium (see Figure S1c)

Strain	Insertion length	position
YS 20.1	460 bp	4 bp upstream of PAM
YS 20.5	456 bp	4 bp upstream of PAM
YS 20.7	118 bp	2 bp upstream of PAM
YS 20.9	236 bp	2 bp upstream of PAM
YS 20.11	620 bp	2 bp upstream of PAM
YS 20.15	637 bp	2 bp upstream of PAM
**Strain**	**Deletion length**	**position**
YS 20.2	195 bp	101 bp~295 bp after *pyrG* start codon
YS 20.6	195 bp	101 bp~295 bp after *pyrG* start codon
YS 20.14	195 bp	101 bp~295 bp after *pyrG* start codon

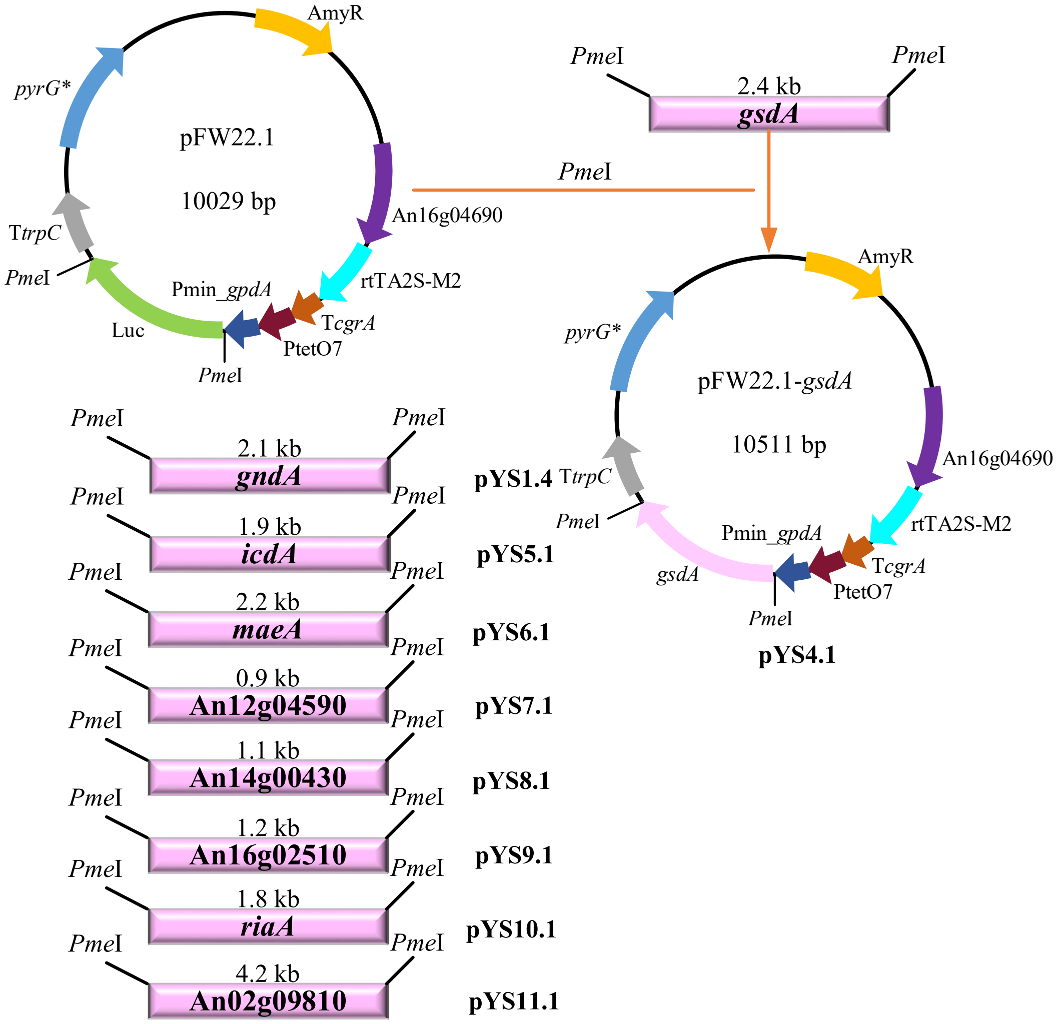

Figure S2 Plasmid construction strategy used for gene overexpression. Vector pFW22.1 was used as an entry vector and digested with *Pme*I. Genes of interest were amplified with primers containing overlapping regions to the plasmid backbone.

Table S2 Positive transformants using AB4.1 as recipient strain verified by Southern analysis; a part of the *pyrG* gene was used as a probe and expected sizes of probed fragments are shown in the corresponding column.

Gene	Strain	*Xba*I	*Nhe*I	*Pvu*I	*Nco*I
**OE *gsdA***	YS7.4, 7.12	7.7 kb,<br>6.7 kb			8.4 kb,<br>3.4 kb
**OE *gndA***	YS9.9, 9.14<br>9.17	7.6 kb,<br>6.7 kb	8.5 kb,<br>7.2 kb		
**OE *icdA***	YS10.6	6.7 kb,<br>5.5 kb	8.5 kb,<br>7.0 kb		
**OE An14g00430**	YS11.8, 11.9		8.5 kb,<br>6.2 kb	9.4 kb,<br>3.8 kb	
**OE *maeA***	YS12.16	7.5 kb,<br>6.7 kb	8.5 kb,<br>2.9 kb		
**OE An12g04590**	YS13.4, 13.7		8.5 kb<br>5.9 kb	9.2 kb,<br>3.9 kb	
**OE An16g02510**	YS14.4		8.7 kb,<br>3.8 kb		8.5 kb,<br>6.3 kb
**control**		3.9 kb	5.5 kb	5.0 kb	3.1 kb

Southern analysis for OE *gsdA* (YS7) transformants:

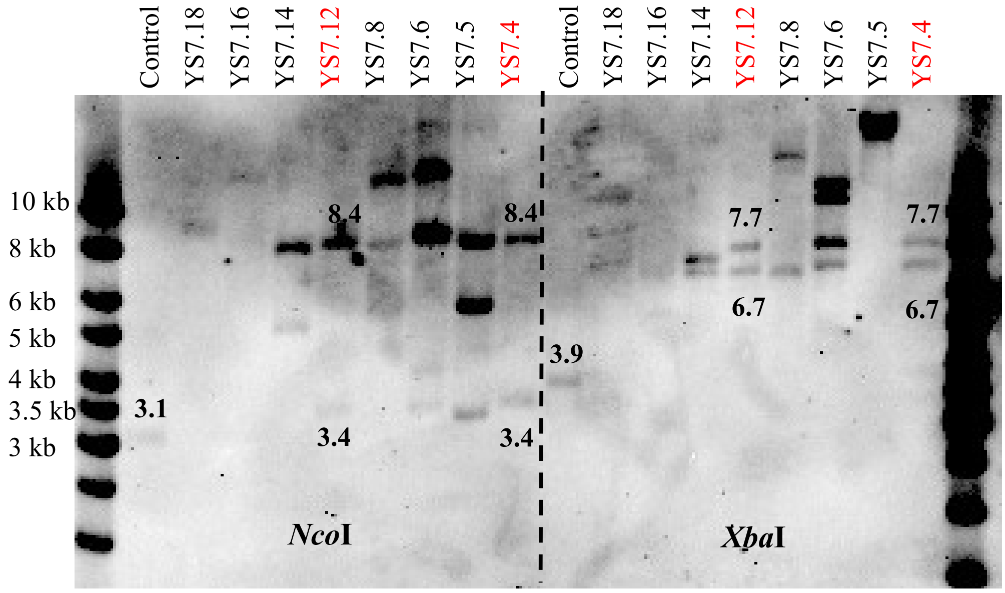

Southern analysis for OE *gndA* (YS9) transformants:

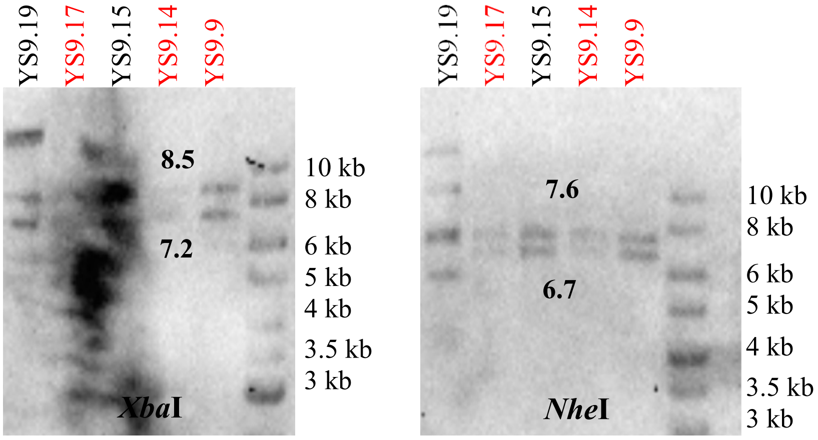

Southern analysis for OE *icdA* (YS10), OE *maeA* (YS12) and OE An12g04590 (YS13) transformants:

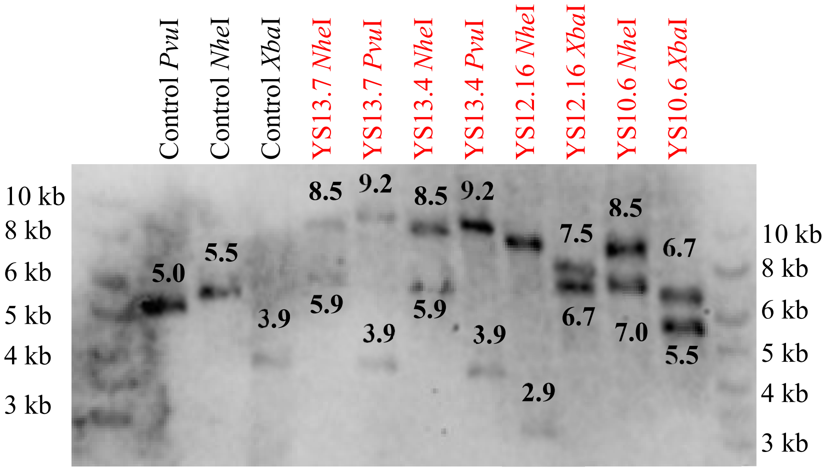

Southern analysis for OE An14g00430 (YS11) transformants:

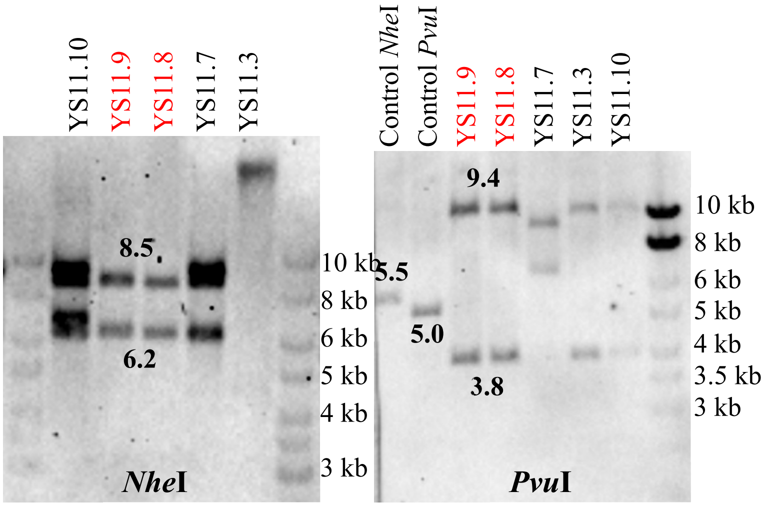

Southern analysis for OE An16g02510 (YS14) transformants:

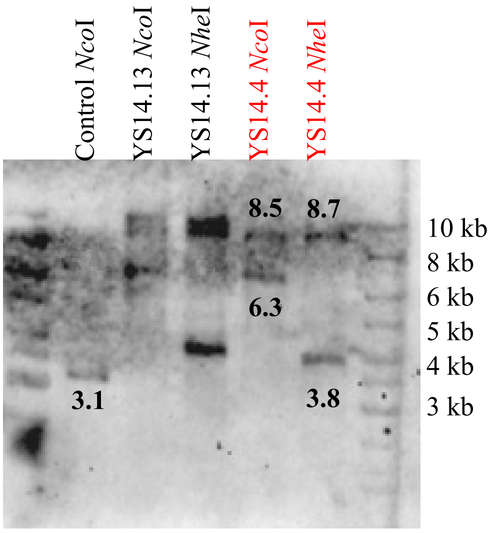

Figure S3 Southern analysis for engineered strains taking AB4.1 as the recipient strain. *pyrG* probe was used to confirm all overexpression strains. Enzymes used for genome digestion and expected signals for single integration are listed in Table S2. Positive transformants are labelled in red.

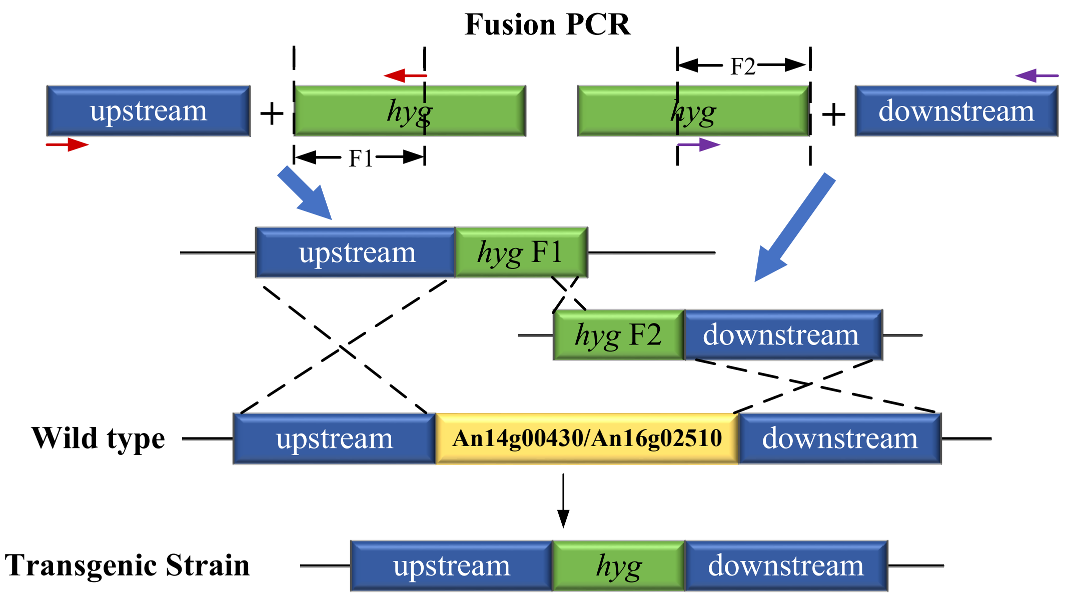

Figure S4 An14g00430 and An16g02510 gene knockout cassettes and homologous recombination strategy. Primer pairs 004305'_fwD/00430_5'_rev and 025105'_fwD/02510_5'_rev were used to amplify the upstream flank of An14g00430 and An16g02510, respectively. In addition, primer pairs 00430_3'_fw/004303'_revD and 02510_3'_fw/025103'_revD were used to amplify the downstream flank of An14g00430 and An16g02510, respectively. Primer pairs 442 hygP6f /443 hygP7r were used to amplify the hygromycin fragment. Split markers were generated by fusion PCR using the primer pairs 00430_5'_fw/445 hygP9r and 02510_5'_fw/445 hygP9r for the first spilt marker and primer pairs 444 hygP8f/00430_3'_rev and 444 hygP8f/02510_3'_rev for the second split marker. The two split markers (1 µg for each) were transformed into YS11.8 (OE An14g00430 strain) or YS14.4 (OE An16g02510 strain). *hyg* represents the hygromycin resistance gene.

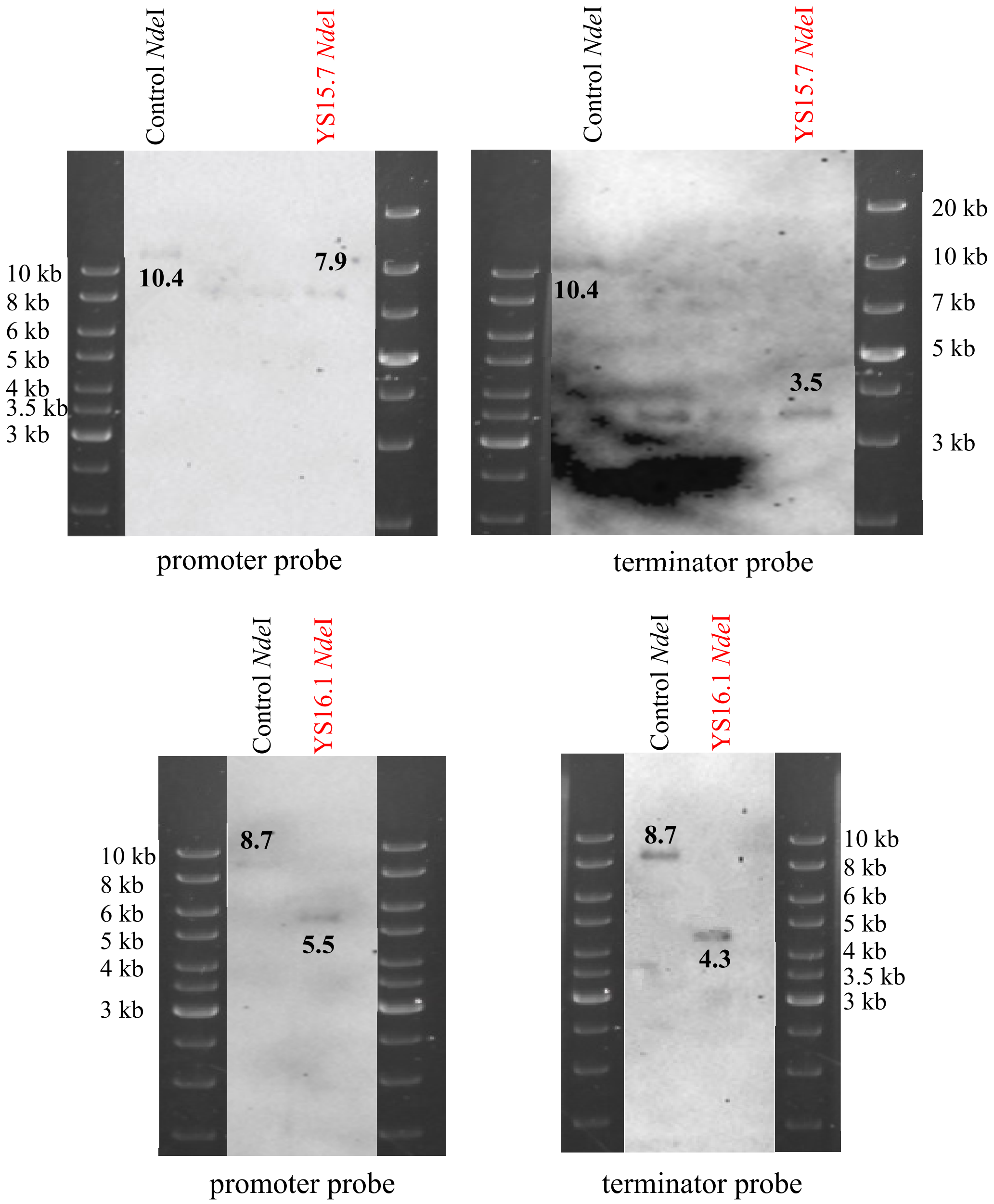

Figure S5 Southern analysis for potential ΔAn16g02510 (YS15) and ΔAn14g00430 (YS16) disruption strains. After digestion with *Nde*I and hybridisation with the An16g02510 promoter probe, a 10.4 kb band was expected for the recipient strain and a 7.9 kb band for knock-out strains. After a digest with *Nde*I and hybridisation with the An16g02510 terminator probe, a 10.4 kb DNA band was expected for the recipient and a 3.5 kb band for ΔAn16g02510 knock-out strains. A digest with *Nde*I and hybridisation with the An14g00430 promoter probe gave a signal at 8.7 kb for the recipient strain and 5.5 kb

for knock-out strains. A digest with *Nde*I and hybridisation with the An16g02510 terminator probe gave a signal at 8.7 kb for the recipient and 4.3 kb for ΔAn14g00430 knock-out strains. Positive transformants are labelled in red.

Table S3 Positive transformants using YS20.2 as recipient strain verified by Southern analysis; a part of the *pyrG* gene was used as a probe and expected sizes of probed fragments are shown in the corresponding column.

Gene	Strain	*Xba*I	*Nhe*I	*Pvu*I	*Nco*I
**OE *maeA***	YS21.13, 21.14	7.5 kb, 6.7 kb	8.5 kb, 2.9 kb		
**OE *gsdA***	YS23.15, 23.20	7.7 kb, 6.7 kb	8.5 kb, 7.5 kb		
**OE *gndA***	YS22.17, 22.20	7.4 kb, 6.7 kb	8.5 kb, 7.2 kb		
**OE An14g00430**	YS24.9		8.5 kb, 6.2 kb	9.1 kb, 3.8 kb	
**OE *icdA***	YS37.4, 37.6 37.7, 37.10	6.7 kb, 5.5 kb	8.5 kb, 7.0 kb		
**OE An16g02510**	YS38.2, 38.4 38.5		8.7 kb, 3.8 kb		8.5 kb, 6.3 kb
**control**		3.9 kb	5.5 kb	5.0 kb	3.1 kb

Southern analysis for OE *mae*A (YS21) and OE *gsd*A (YS23) transformants:

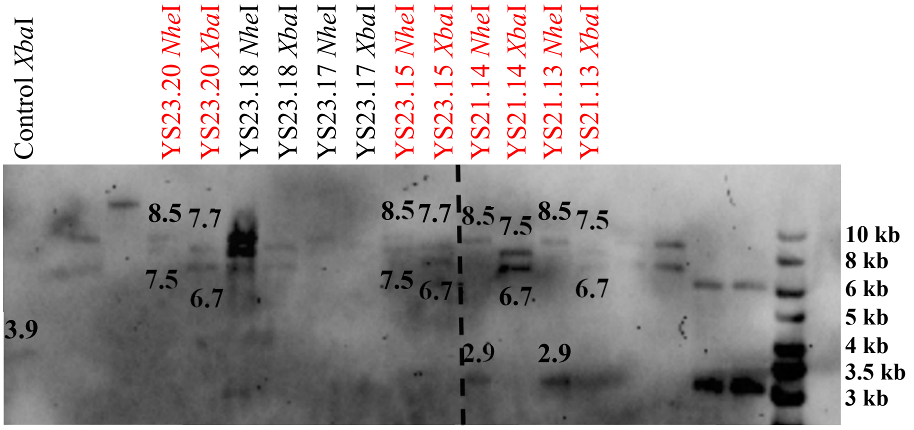

Southern analysis for OE *gndA* (YS22) and OE An14g00430 (YS24) transformants

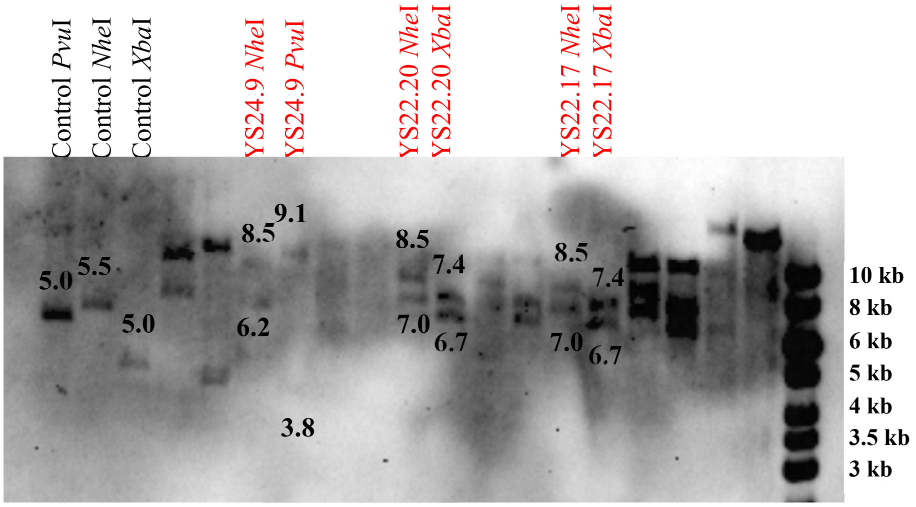

Southern blot analysis for OE *icdA* (YS37) transformants

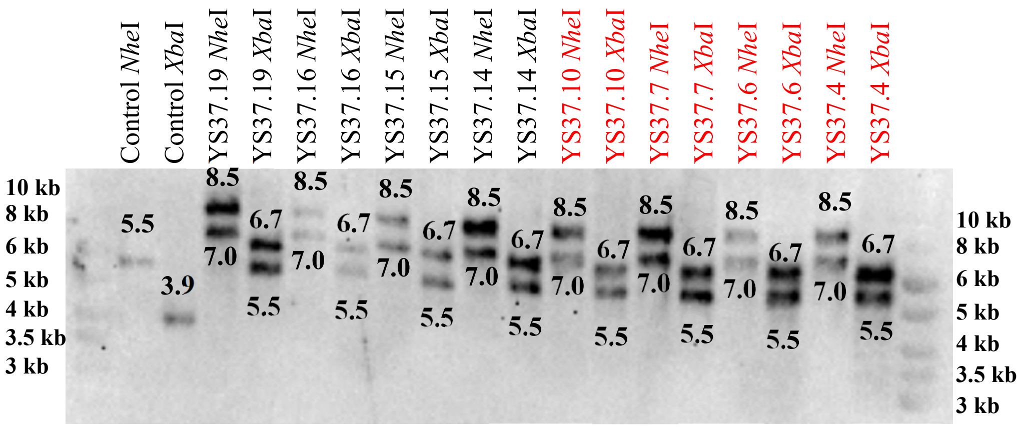

Southern blot analysis for OE An16g02510 (YS38) transformants

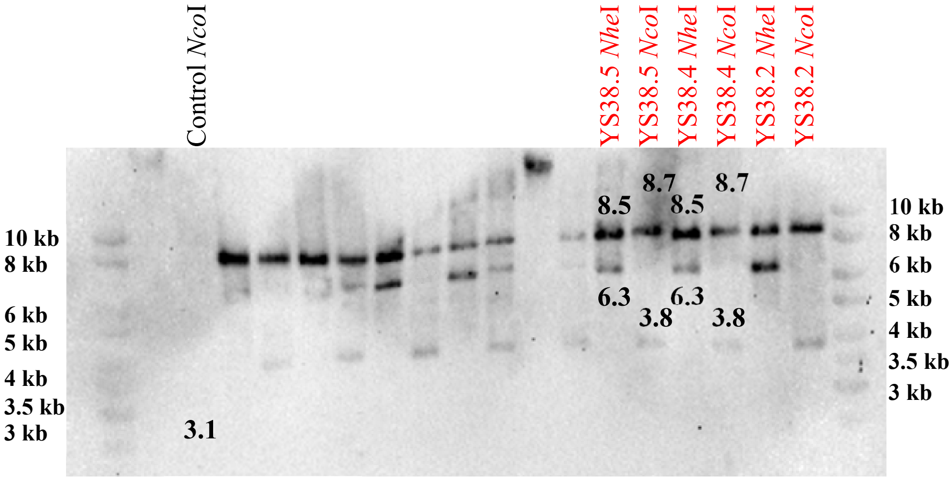

Figure S6 Southern results for engineered strains taking YS20.2 as the recipient strain. The *pyrG* probe was used for the validation of all overexpression strains. Enzymes used for genome digestion, expected signals for single integration are listed in Table S3. Positive transformants are labelled in red.

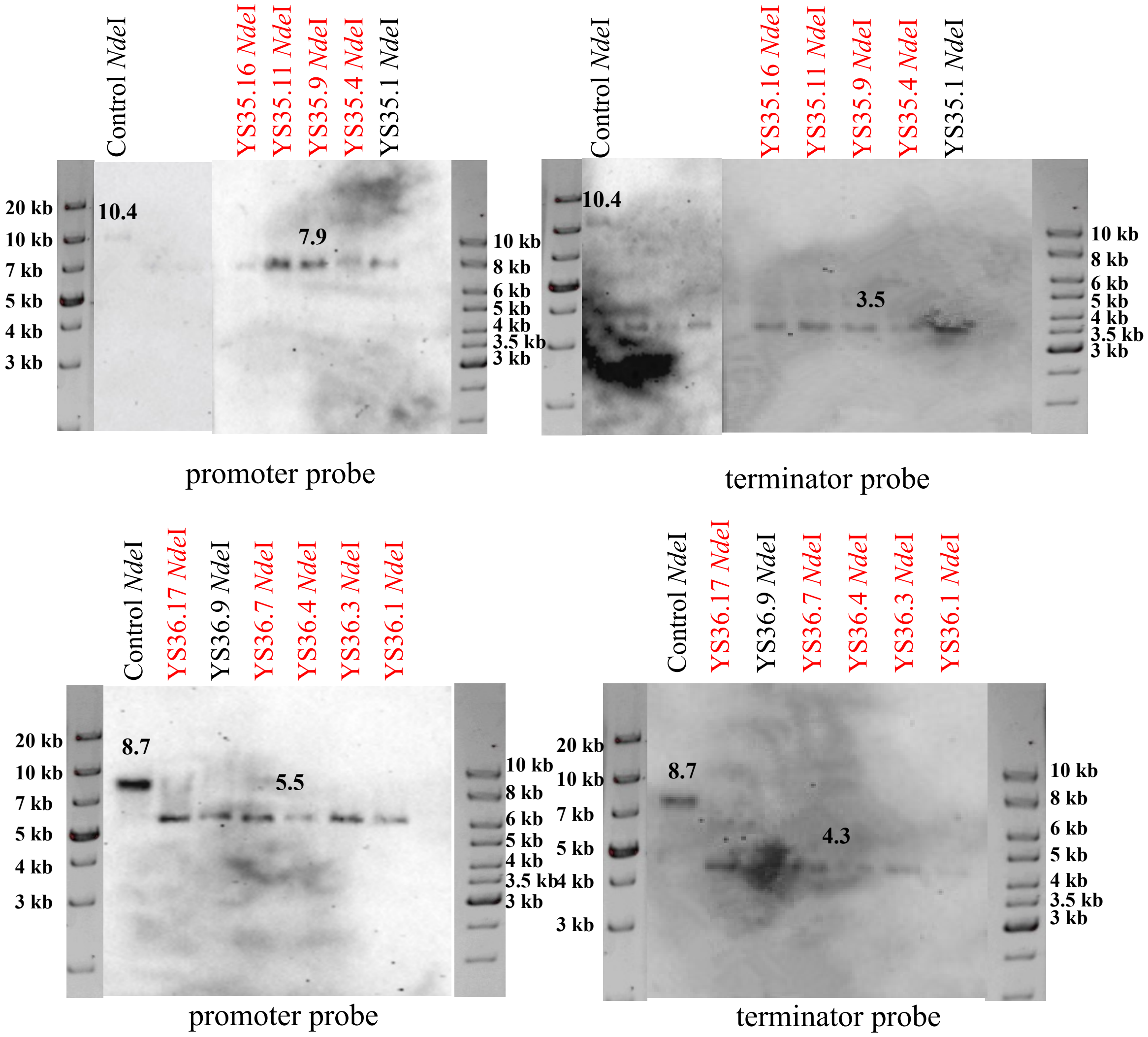

Figure S7 Southern analysis for potential ΔAn16g02510 (YS35) and ΔAn14g00430 (YS36) disruption strains. After digestion with *Nde*I and hybridisation with the An16g02510 promoter probe, a 10.4 kb band was expected for the recipient strain and a 7.9 kb band for knock-out strains. After a digest with *Nde*I and hybridisation with the An16g02510 terminator probe, a 10.4 kb DNA band was expected for the recipient and a 3.5 kb band for ΔAn16g02510 knock-out strains. YS35.4, 35.9, 35.11, 35.16 are positive transformants. A digest with *Nde*I and hybridisation with the An14g00430 promoter probe gave a signal at 8.7 kb for the recipient strain and 5.5 kb for knock-out strains. A digest with *Nde*I and hybridisation with the An16g02510 terminator probe gave a signal at 8.7 kb for the recipient and 4.3 kb for ΔAn14g00430 knock-out strains. YS36.1, 36.3, 36.4, 36.7, and 36.17 are positive transformants. Positive transformants are labelled in red.

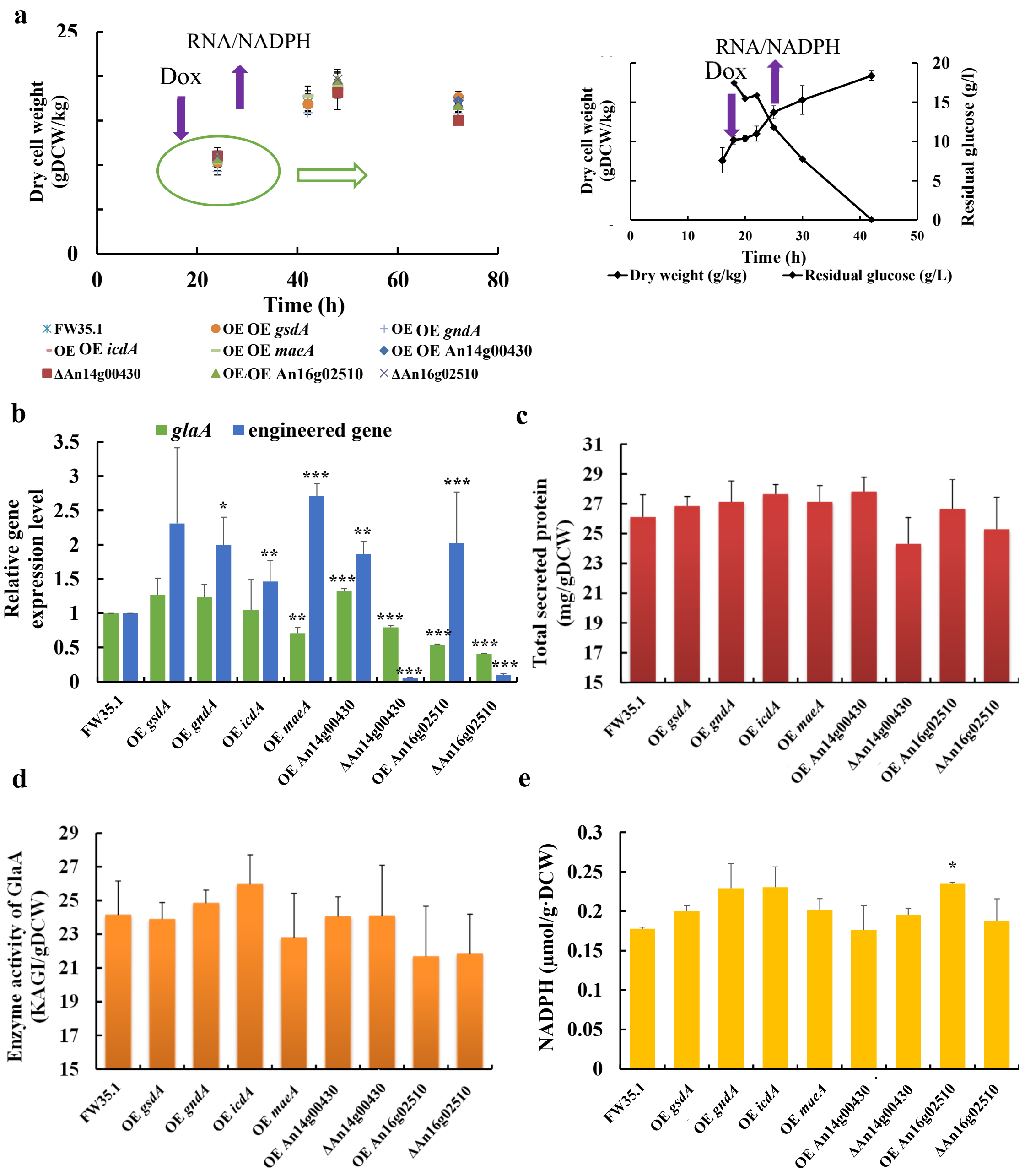

Figure S8 Flask-level fermentation results of all engineered strains in the background of AB4.1 in relation to the control strain FW35.1. (a) Dry cell weight, the addition of doxycycline and samples taken for RNA isolation and NADPH determination are indicated with a purple arrow. The right plot presents more details of the strain growth from 16 h to 42 h; (b) Relative gene expression level of *glaA* and the engineered genes in comparison to the control strain FW35.1; (c) Total secreted protein per gram biomass at 72 h after inoculation; (d) Enzyme activity of GlaA per gram biomass at 72 h after inoculation; (e) Intracellular NADPH level in the exponential phase. All experiments were conducted in

biological quadruplicates. Total secreted protein, enzyme activity of GlaA, and NADPH level were normalized by dry cell weight (DCW). Significance values were calculated with the two-tailed *t*-test with independent variables (**p*<0.05, ***p*<0.01, ****p*<0.001). FW35.1 is the control. OE represents overexpression

Table S4 Summary of flask-level results of all engineered strains compared to AB4.1 or B36. All parameters were measured in biological quadruplicate.

Gene	Strains	Relative expression level of *glaA*	Relative expression Level of engineered genes	Relative NADPH	Relative total secreted protein	Relative enzyme activity of GlaA
**OE *gsdA***	YS7.4	1.3	2.3	12.4%	4.0%	-1.0%
	YS23.20	1.6	2.3	29.5%	7.4%	**, 9.7%
**OE *gndA***	YS9.9	1.2	*, 2.0	*,28.8%	2.9%	2.9%
	YS22.17	***, 2.4	*, 2.2	28.0%	12.8%	***, 17.7%
**OE *icdA***	YS10.6	1.0	**, 1.5	29.5%	6.0%	7.5%
	YS37.6	1.1	***, 1.6	12.1%	12.8%	4.1%
**OE *maeA***	YS12.16	**, 0.7	***, 2.7	13.3%	4.0%	-5.6%
	YS21.14	**, 1.6	**, 1.4	30.4%	*, 11.6%	11.8%
**OEAn14g00430**	YS11.8	***, 1.3	**, 1.9	-1.0%	6.6%	-0.4%
	YS24.9	*, 1.4	2.6	37.5%	3.6%	-0.9%
**ΔAn14g00430**	YS16.1	***, 0.8	***, 0.1	9.9%	-6.9%	-0.3%
	YS36.3	0.9	***, 0.1	11.9%	13.5%	9.5%
**OEAn16g02510**	YS14.4	***, 0.5	2.0	*,32.0%	2.1%	-10.3%
	YS38.2	0.9	***, 4.0	11.1%	2.9%	-5.6%
**ΔAn16g02510**	YS15.7	***, 0.4	***, 0.1	5.4%	-3.1%	-9.5%
	YS35.4	0.7	***, 0.02	-9.6%	-1.2%	-11.6%

Significance values were calculated with the two-tailed *t*-test with independent variables (**p*<0.05, ***p*<0.01, ****p*<0.001). The upper row within one block represents relative values from the engineered strain compared to the reference strain FW35.1, and the lower row are relative values compared to B36. OE represents overexpression

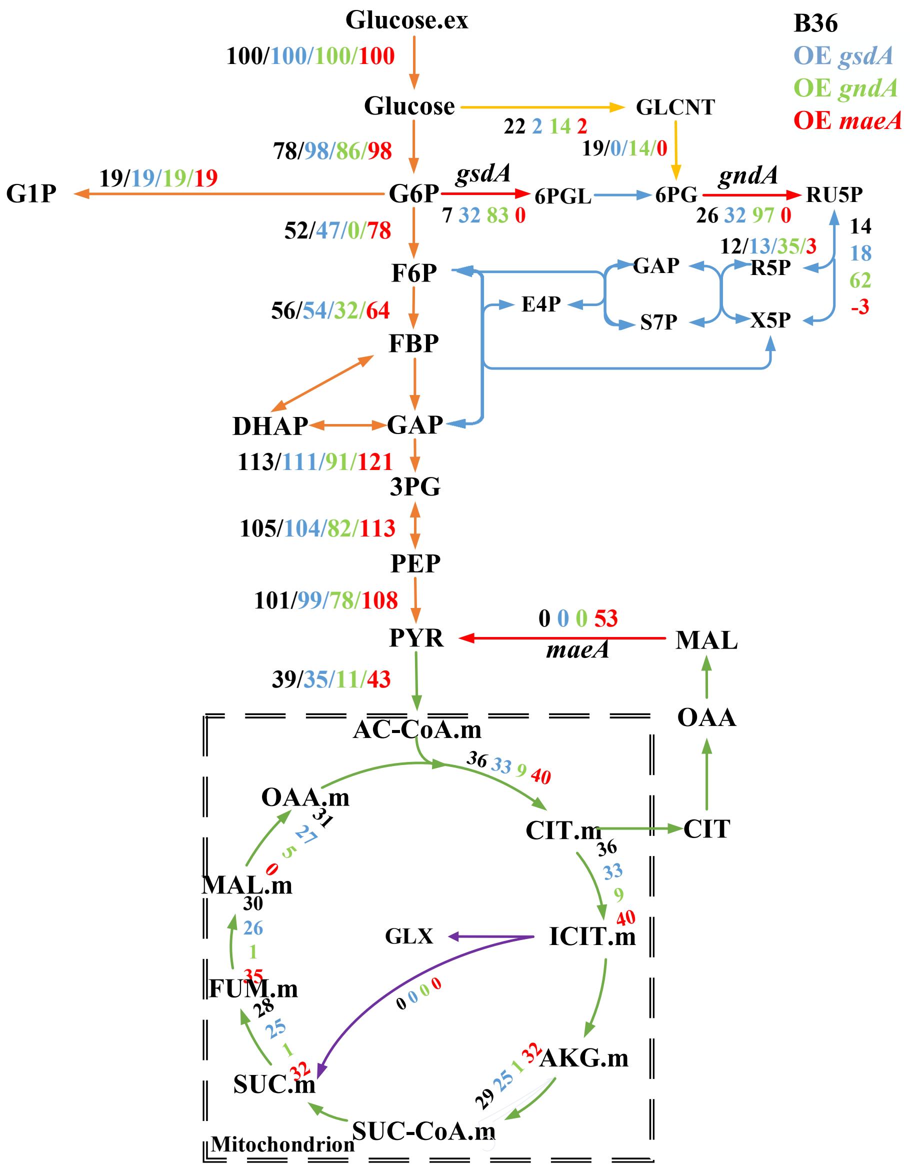

Figure S9 ***In vivo*** **flux distribution of EMP, PPP, and TCA cycle in B36 and three engineered strains at steady state predicted by iHL1210**

Table S5 Pool sizes of intracellular amino acids of *A. niger* B36 (control) and three optimized strains at steady state. The unit is μmol/g_{DCW}. All amino acids were measured in technical triplicate.

Name	B36	OE *gsdA*	OE *gndA*	OE *maeA*
Gln	155.52±2.50	204.01±27.37	153.52±18.31	202.97±17.40
Glu	91.54±1.23	101.21±5.12	87.90±6.18	110.50±12.41
Leu	45.21±4.96	54.24±18.43	39.88±5.73	51.69±5.87
Asp	23.85±0.70	33.88±6.69	26.36±7.53	32.58±5.52
Ala	14.38±0.74	15.41±3.13	12.17±2.41	25.08±5.58
Ser	12.45±1.36	11.44±1.12	10.20±1.84	14.95±3.74
Orn	5.37±0.12	7.52±0.79	5.48±0.92	11.48±3.36
Thr	6.17±0.37	3.81±0.34	5.47±0.27	8.46±0.79
Lys	3.38±0.23	4.32±0.86	3.12±0.44	5.02±0.66
Asn	3.00±0.04	5.30±1.08	3.09±0.67	4.61±1.34
Gly	2.50±0.81	2.29±0.51	1.91±0.29	3.28±0.60
Val	1.11±0.05	1.45±0.31	0.97±0.18	1.80±0.34
Pro	0.78±0.041	0.97±0.19	0.63±0.03	1.21±0.37
His	0.72±0.051	2.86±0.53	1.13±0.26	n.a *
Cys	0.67±0.037	0.38±0.026	0.54±0.038	1.12±0.15
Ile	0.43±0.058	0.69±0.26	0.35±0.031	0.61±0.14
Phe	0.38±0.053	0.45±0.15	0.27±0.033	0.43±0.063
Tyr	0.28±0.037	0.38±0.095	0.25±0.034	0.45±0.077
Met	0.09±0.044	0.20±0.13	0.07±0.021	0.10±0.021
Total	367.68	450.73	353.86	477.03

n.a The His abundance in OE *maeA* was detected incorrectly, thus it didn't show here.

Table S6 Pool sizes of organic acids, sugar phosphates, and energy substrates for *A. niger* B36 and three optimized strains at steady state. The unit is µmol/gDCW. All metabolites were measured in technical triplicate.

Name	B36	OE *gsdA*	OE *gndA*	OE *maeA*
Organic acids				
PYR	0.51±0.01	0.54±0.02	0.75±0.03	0.54±0.006
FUM	3.29±0.5	1.72±0.14	3.32±0.2	5.44±0.5
SUC	1.62±0.11	1.56±0.24	1.48±0.24	1.33±0.17
AKG	0.99±0.14	0.47±0.18	1.06±0.15	0.82±0.09
OAA	0.09±0.012	0.04±0.004	0.03±0.01	0.13±0.01
MAL	2.84±0.2	3.68±0.47	2.6±0.29	2.7±0.1
CIT	30.2±1.6	26.22±0.7	25.77±0.47	27.54±0.57
3PG	2.33±0.4	2.02±0.47	1.41±0.31	3.85±0.22
Sugar phosphates				
F6P	3.56±0.44	2.57±0.11	2.63±0.12	4.04±0.15
G6P	3.27±0.34	2.27±0.13	2.31±0.2	2.92±0.1
G3P	0.14±0.02	0.12±0.01	0.13±0.016	0.15±0.016
E4P	1.59±0.12	3.07±0.21	2.67±0.4	4.4±1.28
6PG	0.49±0.06	0.6±0.07	0.29±0.04	0.45±0.11
S7P	2.00±0.11	1.77±0.08	1.63±0.24	2.08±0.45
PEP	0.13±0.015	0.18±0.03	0.09±0.01	0.255±0.07
FBP	1.08±0.15	0.61±0.2	1.31±0.19	1.36±0.4
R5P	0.5±0.08	0.32±0.04	0.29±0.02	0.54±0.12
Energy substrates				
AMP	0.087±0.018	0.056±0.024	0.081±0.030	0.087±0.0075
ATP	4.12±0.25	5.80±0.01	3.25±1.22	5.68±1.96
ADP	0.582±0.042	0.55±0.034	0.76±0.26	0.63±0.31
ATP/AMP	49.17±13.5	110.28±60.0	44.89±22.75	64.87±20.91
Energy charge	0.92±0.01	0.94±0.013	0.88±0.06	0.94±0.011
NAD+	3.65±0.33	3.308±1.31	6.39±0.49	9.87±2.86
NADH	0.41±0.047	0.40±0.091	0.27±0.093	0.42±0.072
NADH/NAD+	0.11±0.022	0.14±0.067	0.043±0.017	0.043±0.007

Table S7 Primers used in this study

Primer	Sequence
Primers used for cloning overexpression cassettes	
gsdA-F	acagctaccccgcttgagcagacatcaccgtttAAACATGGCCAGCACAATAGCACGCA
gsdA-R	gttaagtggatcccggtcggcatctactgtttaaacTTACAGACGGTTGGGGGTGGAAG
gndA-F	acagctaccccgcttgagcagacatcaccgtttAAACATGGCTGACCAAGCTGTGTAAG
gndA-R	gttaagtggatcccggtcggcatctactgtttaaacTTAGGCAATGTAGGTGGACGCAG
icdA-F	gctaccccgcttgagcagacatcaccgtttAAACATGGCTACCGAAATCTCCAAGATCA
icdA-R	cgttaagtggatcccggtcggcatctactgtttaaacTTAGAGGCGGGACTTGAGGTTG
maeA-F	aacagctaccccgcttgagcagacatcaccgtttAAACATGGCTCGATTTCCCGCTCAG
maeA-R	taagtggatcccggtcggcatctactgtttaaacTCACAGCTTAGAGTTCTTCTCAGCC
An14g00430-F	gctaccccgcttgagcagacatcaccgtttAAACATGCTATTTTCATCATTGCGCTCTC
An14g00430-R	aagtggatcccggtcggcatctactgtttaaacTCAGTAATCATAGAAACCCTTTCCAG
An12g04590-F	cagctaccccgcttgagcagacatcaccgtttAAACAGTAACCAATAAAGATGCCCCCA
An12g04590-R	ttaagtggatcccggtcggcatctactgtttaaacTGCCGTATACATAGACTACCTTGC
An16g02510-F	ctaccccgcttgagcagacatcaccgtttAAACATGGCTCAAGATTACAAGTTTGAAGG
An16g02510-R	ttaagtggatcccggtcggcatctactgtttaaacTCACTGCTCATTCACCAGCACGTA
Primers used to the construction of ΔAn14g00430 or ΔAn16g02510 knockout cassette	
00430_5'_fw	TCCGGAGGAGATAAAGCAGAAGT
00430_5'_rev	caattccagcagcggcttAATGCCAATTTGGGCACGAA
004305'_fwD	GTTGGAGCACATGGTCAGAA
00430_3'_fw	cacggcacaattatccatcgCTCTGTATCTATCCAAGCGAACGT
004303'_revD	GTCATCCTTCCCGTGCTAACA
02510_5'_fw	TGGAATGTCAGCCCGGAAATAT
02510_5'_rev	caattccagcagcggcttGAATGAGTCAGTGGTCGGACA
025105'_fwD	AGCGGTCAATCGAATTACCGA
02510_3'_fw	cacggcacaattatccatcgCGATGTATGGAGTTGGGTGATGT
02510_3'_rev	GGATGAAGACGGAGACATCCT
025103'_revD	GTCCTGGCAGTCATCAAAGGTA

Primer	Sequence
442 hygP6f	aagccgctgctggaattgGGCTCTGAGGTGCAGTGGAT
443 hygP7r	cgatggataattgtgccgtgTTGGGTGTTACGGAGCATTCA
444 hygP8f	AAAGTTCGACAGCGTCTCC
445 hygP9r	GGCGTCGGTTTCCACTATC
Primers used for confirming the integration at the *pyrG* locus	
392pBlueUniversal	GATTAAGTTGGGTAACGCCAGGGT
393*PyrG*Locus	TGTTGAATGGCAACGCTGTCG
Primers used for amplifying *pyrG* probe	
AB*pyrG*P3	TCTCGCGCAGAAGCACAACT
AB*pyrG*P5	GCAGCCTGCACCGGATCG
Primers used for amplifying *pyrG* region after the CRISPR editing	
542	ATTTCTCAGTAGTATCCGCGCACG
169	GCAGCCTGCACCGGATCG
Primers used for quantitative real-time PCR	
RT-gndA-FW	AGGAGATTGCCGATGTGTTC
RT-gndA-rev	CCGTCGTTGTCGTTGAAGTA
RT-gsdA-FW	CCCTACCAAGGAAATCGAAGAG
RT-gsdA-rev	AGGTGCTTGTTGAGGTTGAT
RT-icdA-FW	TACGAGAGCACCTACCAGAA
RT-icdA-rev	CACCCTCACTCTTGATCATCTG
RT-maeA-FW	GGTATTGGTCTGGGTACCATTC
RT-maeA-rev	GTAGAGAAGACCACGCTCAATC
RT-An14g00430-FW	GTTAAGTCGCTGGGTGTGATAG
RT-An14g00430-rev	TGCTTGGGAGTTGTCGATTAG
RT-An12g04590-FW	CTGCATCTGCTGGTGGTAAT
RT-An12g04590-rev	TTCCTCTCCATACCTCGTCTC
RT-An16g02510-FW	GCTGATAAGGTGGTTGCTATCT
RT-An16g02510-rev	GGCTCATCATCCGTAGCAATA

Linker sequences are shown in lowercase, restriction sites are underlined. Primer pairs gsdA-F/gsdA-R, gndA-F/gndA-R, icdA-F/icdA-R, maeA-F/maeA-R, An14g00430-F/An14g00430-R, An12g04590-F/An12g04590-R, An16g02510-F/An16g02510-R were used to amplify the ORF region of *gsdA*, *gndA*, *icdA*, *maeA*, An14g00430, An12g04590 and An16g02510. Primer pairs used for gene deletion are also explained in Figure S4. Primer pairs RT-gndA-FW/RT-gndA-rev, RT-gsdA-FW/RT-gsdA-rev, RT-icdA-FW/RT-

icdA-rev, RT-maeA-FW/RT-maeA-rev, RT-An14g00430-FW/RT-An14g00430-rev, RT-An12g04590-FW/RT-An12g04590-rev, and RT-An16g02510-FW/RT-An16g02510-rev were used to quantify the relative gene expression level of *gsdA*, *gndA*, *icdA*, *maeA*, An14g00430, An12g04590 and An16g02510 through quantitative real-time PCR.

Table S8 Plasmids constructed in this study

Plasmid name	Relevant gene
pYS1.4	An12g12140 （*gndA*）
pYS4.1	An11g02040 （*gsdA*）
pYS5.1	An02g12430 （*icdA*）
pYS6.1	An05g00930（*maeA*）
pYS7.1	An12g04590
pYS8.1	An14g00430
pYS9.1	An16g02510

Table S9 Primers used for *in vitro* sgRNA synthesis

Primer name	Sequence
*pyrG*sgRNA1_fw	ATGTAATACGACTCACTATAGGTAACGGTGATACCGGACTGCGTTTTAGAGCTAGAAATAGCAAGT
*pyrG*sgRNA2_fw	ATGTAATACGACTCACTATAGGCGGCGGTACCCTCCGTATCTGTTTTAGAGCTAGAAATAGCAAGT
sgRNAstd_rev	AGCACCGACTCGGTGCCACTTTTTCAAGTTGATAACGGACTAGCCTTATTTTAACTTGCTATTTCTAGCTCTAAAAC

* Red letters represent the specific 20 bp protospacer sequences. ATGTAATACGACTCACTATAGG is T7 promoter sequence.
